# Abnormal cell sorting and altered early neurogenesis in a human cortical organoid model of Protocadherin-19 clustering epilepsy

**DOI:** 10.3389/fncel.2024.1339345

**Published:** 2024-04-04

**Authors:** Wei Niu, Lu Deng, Sandra P. Mojica-Perez, Andrew M. Tidball, Roksolana Sudyk, Kyle Stokes, Jack M. Parent

**Affiliations:** ^1^Department of Neurology, University of Michigan, Ann Arbor, MI, United States; ^2^Department of Biological Sciences, University of Toledo, Toledo, OH, United States; ^3^Department of Rehabilitation, the Second Xiangya Hospital, Central South University, Changsha, Hunan, China; ^4^Michigan Neuroscience Institute, University of Michigan, Ann Arbor, MI, United States; ^5^VA Ann Arbor Healthcare System, Ann Arbor, MI, United States

**Keywords:** PCDH19-clustering epilepsy, neurodevelopment, CRISPR/CAS9 genome editing, brain organoid, mosaicism, random X-chromosome inactivation, Protocadherin-19

## Abstract

**Introduction:**

Protocadherin-19 (*PCDH19*)-Clustering Epilepsy (PCE) is a developmental and epileptic encephalopathy caused by loss-of-function variants of the *PCDH19* gene on the X-chromosome. PCE affects females and mosaic males while male carriers are largely spared. Mosaic expression of the cell adhesion molecule PCDH19 due to random X-chromosome inactivation is thought to impair cell–cell interactions between mutant and wild type *PCDH19*-expressing cells to produce the disease. Progress has been made in understanding PCE using rodent models or patient induced pluripotent stem cells (iPSCs). However, rodents do not faithfully model key aspects of human brain development, and patient iPSC models are limited by issues with random X-chromosome inactivation.

**Methods:**

To overcome these challenges and model mosaic *PCDH19* expression *in vitro*, we generated isogenic female human embryonic stem cells with either HA-FLAG-tagged *PCDH19* (WT) or homozygous *PCDH19* knockout (KO) using genome editing. We then mixed GFP-labeled WT and RFP-labeled KO cells and generated human cortical organoids (hCOs).

**Results:**

We found that PCDH19 is highly expressed in early (days 20–35) WT neural rosettes where it co-localizes with N-Cadherin in ventricular zone (VZ)-like regions. Mosaic PCE hCOs displayed abnormal cell sorting in the VZ with KO and WT cells completely segregated. This segregation remained robust when WT:KO cells were mixed at 2:1 or 1:2 ratios. PCE hCOs also exhibited altered expression of PCDH19 (in WT cells) and N-Cadherin, and abnormal deep layer neurogenesis. None of these abnormalities were observed in hCOs generated by mixing only WT or only KO (modeling male carrier) cells.

**Discussion:**

Our results using the mosaic PCE hCO model suggest that PCDH19 plays a critical role in human VZ radial glial organization and early cortical development. This model should offer a key platform for exploring mechanisms underlying PCE-related cortical hyperexcitability and testing of potential precision therapies.

## 1 Introduction

*PCDH19*-Clustering Epilepsy (PCE), formally known as developmental and epileptic encephalopathy-9 (DEE9), is caused by pathogenic variants in Protocadherin-19 (*PCDH19*), an X-chromosome gene that undergoes random X-inactivation (RXI) (Dibbens et al., 2008; Kolc et al., 2019). PCE has an unusual X-linked inherence pattern, as it exclusively affects females and mosaic males while male carriers are largely spared (Depienne et al., 2009). *De novo* germline pathogenic variants in females are also common (Depienne and LeGuern, 2012). PCE is one of the more common genetic DEEs with an estimated incidence of 1 per 20,600 live born females (Symonds et al., 2019). Many females with PCE develop seizures in infancy, often evoked by fevers. Seizures typically occur in clusters that last days or longer. Over 50% of patients have cognitive impairment, intellectual disability (ID), autism spectrum disorder (ASD), and other neuropsychiatric co-morbidities (Kolc et al., 2019, 2020).

PCDH19 is a member of the non-clustered delta-PCDH subfamily of cadherins, a group of cell adhesion molecules (CAMs). It consists of six extracellular cadherin (EC) domains, a transmembrane region (TM), and a cytoplasmic C-terminal tail with two conserved domains (CM1 and CM2) (Cooper et al., 2016). PCDH19 molecules from adjacent cells form a homophilic “forearm handshake” binding through the EC domain (Cooper et al., 2016). The EC domain is also important for mediating interactions with other cadherin proteins including N-Cadherin (NCAD) (Biswas et al., 2010). PCDH19 is predominantly expressed in the brain and thought to regulate neuronal migration, circuit formation, and synaptic plasticity/maintenance through both cell–cell interactions and signal transduction pathways (Kim et al., 2011). Over 150 pathologic variants of *PCDH19*, including both missense and frameshift mutations, have been identified (Kolc et al., 2019). Pathogenic variants are predominantly located in the first exon that encodes the EC domains that are required for the intercellular adhesive activity of the protein (Marini et al., 2012), although complete *PCDH19* deletions also occur, supporting a loss-of-function (LoF) mechanism (Kolc et al., 2019).

How heterozygous PCDH19 LoF leads to clinical PCE phenotypes remains uncertain. One compelling hypothesis is that tissue mosaicism from RXI in heterozygous females leads to impaired interactions during brain development between cells expressing wild type (WT) *PCDH19* and those expressing mutant *PCDH19*, a phenomenon known as “cellular interference” (Depienne et al., 2009). This theory is supported by the identification of PCE in males mosaic for pathogenic *PCDH19* variants, while hemizygous males carrying only mutant *PCDH19* do not develop PCE (Depienne and LeGuern, 2012). Recent studies using *in vivo* rodent models have made progress on understanding this “cellular interference” mechanism. One study in a mouse PCE model provided evidence to support the idea that the PCDH19-NCAD cis-complex in WT neurons cannot trans-synaptically bind NCAD on *PCDH19* null cells (Hoshina et al., 2021). Multiple *Pcdh19* heterozygous knockout (KO) mouse PCE models have shown abnormal cell sorting with wide columnar cortical regions consisting exclusively of WT Pcdh19-expressing cells segregated from columns of Pcdh19 KO cells (Hayashi et al., 2017; Pederick et al., 2018; Hoshina et al., 2021). However, human brain development differs considerably from that of rodent in many critical aspects including larger brain volume, greater diversity of cell types, and an expanded subventricular zone (SVZ) (Rakic, 1995b; Miller et al., 2019). Moreover, existing PCE mouse models do not develop spontaneous seizures. A significant gap therefore remains in understanding both the precise function of PCDH19 during human cortical development and how mosaic expression of *PCDH19* leads to PCE.

Patient-derived iPSCs that carry patient-specific variants are appealing models for the understanding of cellular processes that lead to X-linked neurological disorders (Marchetto et al., 2010; Ananiev et al., 2011). Studies of PCE patient iPSCs (Homan et al., 2018; Borghi et al., 2021; Alaverdian et al., 2023) or shRNA-mediated PCDH19 knockdown in control iPSCs (Giansante et al., 2023) have been used to model PCE *in vitro*. In these human iPSC models, however, *PCDH19* WT and mutant cells were observed to mix extensively without evidence of cell segregation. Female PCE patient iPSC studies are difficult to interpret due to unexpected X chromosome skewing (Pomp et al., 2011; Mekhoubad et al., 2012; Tomoda et al., 2012) and challenges in determining the ratios of cells expressing WT vs. mutant *PCDH19* after RXI in any given experiment. To circumvent the issue of RXI with iPSC culture, we created a novel *in vitro* system to model the mosaic expression of PCDH19 that results in robust cell segregation phenotypes. Separation of *PCDH19* KO and WT neural stem cells appeared in our mosaic PCE cortical organoid model, but not in cultures of exclusively WT or KO cells. Segregated mosaic PCE model cultures also exhibited aberrant development of deep-layer cortical neurons. In addition to identifying robust PCE pathogenic phenotypes, our approach provides a powerful platform to advance understanding of PCDH19 function during human cortical development and to identify precision PCE therapies using human cells.

## 2 Materials and methods

### 2.1 Stem cell culture

Female H9 hESC lines (WA09; WiCell) were maintained in feeder-free conditions on Geltrex-coated plates in TeSR™-E8™ maintenance medium (STEMCELL technologies, Cat#05940) or Stemflex medium (ThermoFisher, Cat # A3349401) at 37°C with controlled humidity and 5% CO_2_. Cells were routinely passaged with 0.8 mM EDTA when the cultures reached ~80% confluency, and 10 μM ROCK inhibitor Y27632 was added to TeSR™-E8™ medium on the day of passaging. No ROCK inhibitor was used when cells were passaged in StemFlex medium. hESC lines were maintained similarly. All hESC work was approved by the University of Michigan Human Pluripotent Stem Cell Research Oversight (HPSCRO) committee.

### 2.2 CRISPR/Cas9 genome editing

We followed an established CRISPR gene editing protocol (Ran et al., 2013) to generate both *PCDH19* HA-FLAG tagged H9 hESC lines and isogenic homozygous KO lines. A short guide RNA (sgRNA) targeting the 3’-UTR region of *PCDH19* and a donor plasmid was used for in-frame tagging of the *PCDH19* locus with HA-FLAG sequences (5’-TAC CCA TAC GAT GTT CCA GAT TAC GCT GAC TAC AAA GAC GAT GAC GAC AAG-3′). The sgRNA sequence for HA-FLAG tagging was 5’-GCCACTCAAGAACCAACCAT-3′. The sgRNA was designed using the UCSC genome browser on Human Feb. 2009 (GRCh37/hg19) Assembly^1^ and was cloned into pSpCas9(BB)-2A-GFP vector (PX458; Addgene #48138). The HA-FLAG sequence is 5’-TAC CCA TAC GAT GTT CCA GAT TAC GCT GAC TAC AAA GAC GAT GAC GAC AAG −3′. The repair DNA template that contains the desired HA-FLAG sequence and ~ 1 kb sequences on each side of a stop codon was cloned into pUC19 plasmid (ThermoFisher, Cat# C40005). The CAS9-sgRNA vector and donor template were delivered to H9 hESCs using Mirus Bio TransIT-LT1 Transfection Reagent (Mirus Bio LLC, cat# MIR2304). The primer set to confirm the HA-FLAG tag was: 5’-GCCACTCAAGAACCAACCAT-3′ and 5’-ACAAAGAGTCCCC TGGTGTG-3′. To make homozygous insertions/deletions (indels), we used a sgRNA to target the first exon of the PCDH19 gene. The sgRNA sequence for tagging was 5’-CCACCACGAGTTCGGC AAAG-3′. It was cloned into pSpCas9(BB)-2A-Puro V2.0 vector (PX459, Addgene, #62988). To ensure the purity of each clonal colony, we performed single-cell cloning for two gene edited clones, KO4 and KO9 (Mojica-Perez et al., 2023). The primer set to confirm indels is: 5’-GTCATTGGAGTCGGTCACCT-3′ and 5’-CCAGACTCAGGAAG CTTTGG-3′. We validated both genome edited hESC lines using both Sanger sequencing through the University of Michigan Sequencing Core and a CRISPR next generation sequencing (NGS) platform (MGH CCIB DNA Core; Massachusetts General Hospital).

### 2.3 3-D cortical organoid generation

We first used lentivirus to label *PCDH19* HA-FLAG tagged H9 hESC lines and isogenic homozygous KO lines with GFP and RFP reporters, respectively, before hCO differentiation. Two different KO lines (KO4-7 and KO10-1) were used in the current study. We utilized a new single rosette method, termed SOSR-CO (self-organizing single rostette-cortical organoid) for differentiation of hPSCs into hCOs (Tidball et al., 2023), with a minor modification of using a lower concentration (1 μM) of CHIR99021 treatment between days 6–10. At day −1, stem cell cultures at 80–90% confluency were dissociated to single cells with accutase. To model the mosaic expression of *PCDH19* found in PCE patients (heterozygous females or mosaic males), we mixed GFP-labeled WT with RFP-labeled isogenic KO *PCDH19* female H9 hESCs at a 1:1 ratio as a “PCE mosaic” condition on the initial day of brain organoid differentiation (day −1); we also mixed RFP-labeled WT with GFP-labeled KO cells. Similarly, we mixed cultures of GFP-labeled/RFP-labeled (1:1) KO only or isogenic WT only cells as controls. Viable cells (2 × 10^6^) were plated onto one well of a Geltrex-coated 24-well plate containing TeSR-E8 with 10 μM Y27632. The next day (day 0) a monolayer of cells formed and was cultured with dual-SMAD inhibitor plus WNT inhibitor medium which contained neural maintenance media (3 N) supplemented with 2 μM DMH1, 2 μM XAV939 and 10 μM SB431542 (Tocris Biosciences). 3 N medium contains 50:50 DMEM/F12:Neurobasal with N2 and B27 supplements without vitamin A. On days 1–3, 1 μM cyclopamine was also added. On day 4, the monolayer neuroepithelium sheet was cut into ~125 μm squares with a StemPro EZ passage tool (Gibco #23181010). These fragments were collected and plated onto 100% Geltrex coated 96-well plates. From days 6–9, half medium changes were performed with 3 N medium supplemented with 1 μM CHIR99021. When the spheroids formed and reached a desired size between 250 and 300 μM in diameter (often at day 9–10), the single rosette spheroids were transferred from 96-well plates to a low-attachment U-bottom plate using a Stripper Pipette tool. From days 11–29, cortical spheroids were maintained in 3 N medium with vitamin A, BDNF (20 ng/mL) and NT3 (20 ng/mL). From day 30 onwards, hCOs were transferred to low attachment 24-well plates and maintained in 3 N medium with vitamin A.

### 2.4 Differentiation of hESCs to 2D neural rosettes and excitatory cortical neurons

We modified previously published protocols for differentiating hESCs to excitatory cortical neurons (Shi et al., 2012). At day −1, hESCs at 80% confluency were dissociated to single cells with accutase and were plated onto one well of a Matrigel-coated 12-well plate containing TeSR-E8 with 10 μM Y27632. The next day (day 0) a monolayer of cells formed and was cultured with 3 N medium with dual-SMAD inhibitors. Cells were maintained in neural induction medium with daily medium changes for 10–12 days to form primitive neuroepithelium. At day 12, neuroepithelium was dissociated to clumps with 2 U/mL Dispase (ThermoFisher), and re-plated onto Matrigel-coated 6-well plates in 3 N medium. When the neural epithelium formed rosettes, 3 N medium was supplemented with 20 ng/mL fibroblast growth factor-2 (FGF2; Peprotech) for 4 days for the expansion of NSCs. At ~day 19, rosettes were dissociated to smaller rosettes with Dispase and re-plated on Matrigel-coated 6-well plates to continue neuronal differentiation. Alternatively, rosettes were re-plated on Nunc Lab-Tek III 8-well chamber slides (Thermo Scientific) and fixed for future immunocytochemistry (ICC) studies.

### 2.5 Immunocytochemistry and microscopy

Stem cells were grown on Nunc™ Lab-Tek™ III 8-well chamber slides (Thermo Scientific), and fixed with 4% paraformaldehyde (PFA) for 20 min at room temperature (RT). Cortical organoids were fixed in 4% PFA for 20–30 min at 4°C, cryoprotected overnight with 30% sucrose in phosphate-buffered saline (PBS), and then embedded in Tissue-Tek OCT embedding medium (Sakura Finetek, Torrance, California, United States) and stored at −80°C. A Leica CM1850 cryostat was used to generate 20 μm thick sections that were collected on Superfrost Plus glass slides (Thermo Fisher) and stored at −20°C. For immunostaining, the hCO sections or fixed stem cells were permeabilized with 0.1–0.2% Triton-X 100 for 20 min at RT, and then incubated in blocking buffer (PBS with 0.05% Tween- 20, 5% normal goat serum, and 1% bovine serum albumin (BSA)) for 1 h at RT. The fixed sections or cells were incubated in primary antibody (Supplementary Table S1) overnight in the same blocking buffer at 4°C, washed 4 times in PBS with 0.05% Tween-20 (PBST), and then incubated for 90 min with secondary antibody (Supplementary Table S1). Sections or cells were washed 3 times in PBST and incubated with bisbenzimide for 5 min. After additional PBS washes, coverslips were mounted on slides with Glycergel mounting medium (Agilent Dako, Santa Clara, CA, United States). For immunostaining of fixed whole mount organoids, we extended the incubation time for primary and secondary antibodies to 24 and 48 h at 4°C respectively, and incubated in nuclear counterstain overnight at 4°C before the final mounting step.

Images were acquired on a Leica SP5 upright DMI 6000 confocal microscope and Leica Stellaris confocal imaging system. For neural rosette analysis, images were acquired under a 20x, 40x, or 63x objective and 1 μm step size through the z-plane with the pinhole set at 1 Airy unit. An EVOS FL Auto Imaging System (Thermo Fisher Scientific) or IncuCyte^®^ Live Cell Analysis System (IncuCyte S3 2018C, Essen Bioscience) was used for acquiring the time lapse images of rosette formation.

### 2.6 Image analysis

Confocal images acquired using a Leica SP5 confocal microscope and Leica Stellaris were analyzed using ImageJ (NIH, Bethesda, MD, USA). The individual who performed the analyses was blinded to genotypes. We outlined ventricular zone (VZ)/SVZ-like and cortical plate (CP)-like regions based on the bisbenzimide DNA staining or GFP signals and set them as two regions of interest. The mean intensity of CTIP2 was measured for each ROI of VZ/SVZ or CP region. These values were then normalized to the background signal to obtain relative fluorescence levels for CTIP2. For counting the number of CTIP2+ cells inside VZ/SVZ regions, we set GFP and RFP cells to two separate ROIs, counted the number of cells in each ROI and then normalized by the area of each ROI.

### 2.7 RNA isolation and RT-qPCR

Total RNA was isolated with miRNeasy Mini Kit (Qiagen) according to the manufacturer’s instructions. cDNA was synthesized with the SuperScript™ III First-Strand Synthesis SuperMix kit (Thermofisher). RT-qPCR was carried out with Power SYBR Green PCR Master Mix (Thermofisher, 4367659). Fold changes were calculated using the 2^-ΔΔCt method: ΔΔCt = [(Ct gene of interest - Ct internal control) KO - (Ct gene of interest - Ct internal control) WT], with GAPDH being an internal control. Primer sets to check mRNA levels were: *SCN1B* - 5′- CTGCTGGCCTTAGTGGTCG-3′ and 5′- AAGGTCATCCCATACACGGC-3′; *PCDH19*–5’-CGAGAGC AGGGGACAAGTAG-3′ and 5’-AATGGCGAAGTCAGAACCAC-3′; *NCAD* - 5’-ATTTGGTGATTCTCGGTCCA-3′ and 5’-CTTATTTT GCCCCCAATCCT-3′; *ACTB*- 5′- ACATCTGCTGGAAGGTGGAC-3′ and 5’-CCCAGCACAATGAAGATCAA-3′; *GAPDH* - 5’-ATGTTCG TCATGGGTGTGAA-3′ and 5’-GGTGCTAAGCAGTTGGTGGT-3′.

### 2.8 Immunoblotting

All tissues were lysed in ice-cold whole cell lysis buffer supplemented with 1 × protease and phosphatase inhibitors (Deng et al., 2022). Protein (50–100 μg) was separated on 4–12% gradient Bis-Tris gels (ThermoFisher, NW04125BOX), and transferred onto 0.45-μm polyvinylidene difluoride (PVDF) membranes (Thermofisher, LC2005). The membranes were blocked in 1 × PBS with 5% non-fat dry milk, incubated with primary antibodies (Supplementary Table S1) at 4°C overnight, washed three times with TBS, then incubated with HRP-conjugated secondary antibodies (CST) for 2 h at RT. Signal was detected using Clarity™ and Clarity Max™ Western ECL Blotting Substrates (Bio-Rad) and captured using a ChemiDoc™ Imaging System (Bio-Rad) or the Odyssey system.

### 2.9 Statistical methods

Statistical analyses were performed using Microsoft Excel (Microsoft, Seatle, WA, United States) and/or Prism (GraphPad Software Inc., LaJolla, CA, United States). Prism was used for generating all the statistical graphs in this study. Analyses of RT-qPCR, blotting results, cell segregation and CTIP2 were performed using a Kruskal-Wallis test with Dunn’s multiple comparisons *post hoc* test. Epifluorescence intensity was analyzed using one-way ANOVA with Dunnett’s multiple comparisons test. Graphs are presented as mean ± standard error of the mean. A confidence interval of 95% (*p* < 0.05) was considered statistically significant.

## 3 Results

### 3.1 Generation of PCDH19 tagged and knockout hESCs

**Figure 1:**
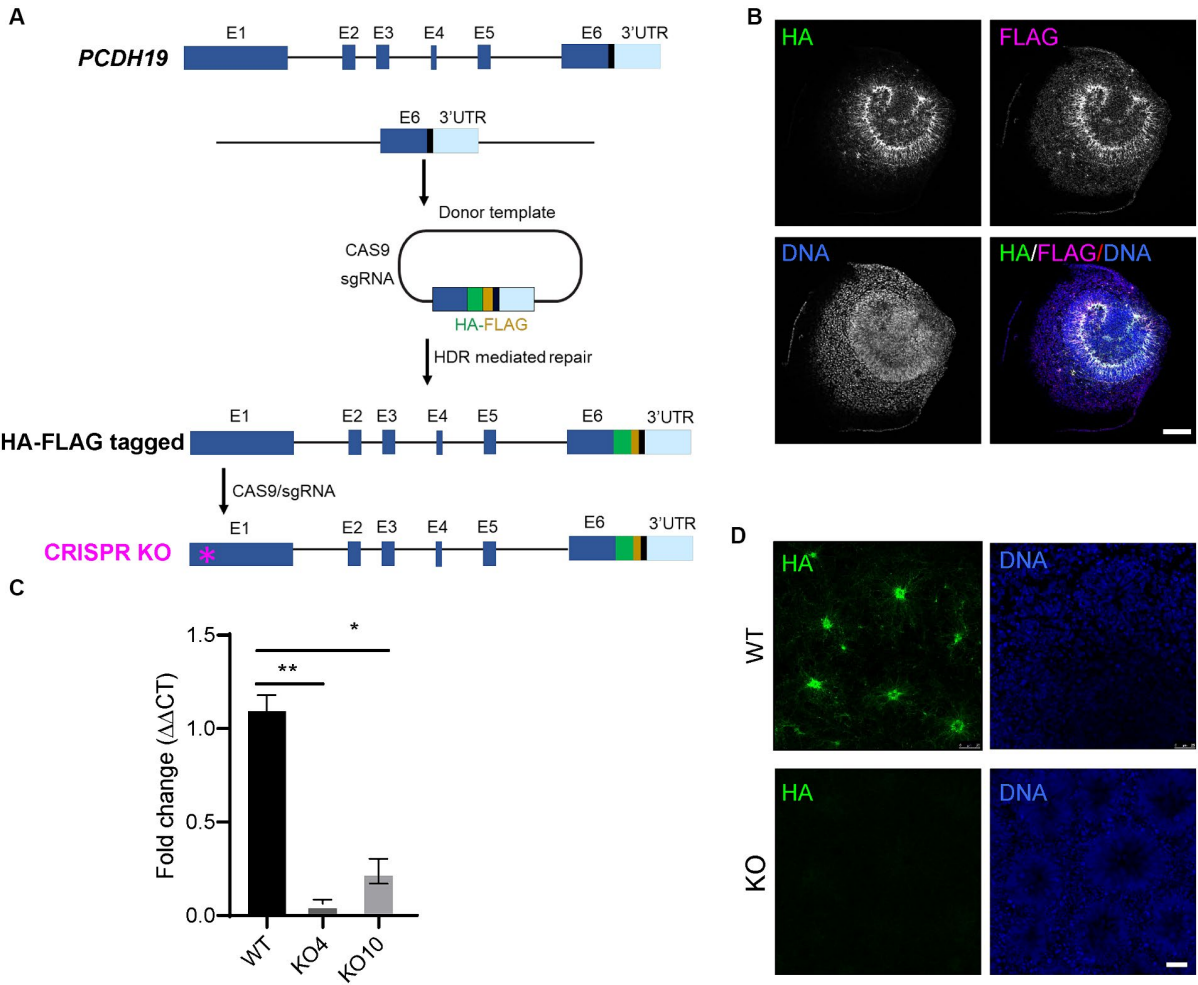
Generation of H9 female human embryonic stem cell (hESC) models of PCE. **(A)** Schema of generating in-frame hemagglutinin (HA)-FLAG tagged *PCDH19* and *PCDH19* KO using CRISPR/CAS9 genome editing. To make HA-FLAG tagged *PCDH19* hESCs (i.e., WT), a sgRNA was used to target to the 3’-UTR region of *PCDH19*, and a donor plasmid containing a HA-FLAG DNA sequence and two 1 kb DNA sequences flanking the stop codon of *PCDH19* was utilized to provide the homology directed repair (HDR) template. To make isogenic *PCDH19* KO cells (i.e., KO), a sgRNA was used to target the 1st exon of the HA-tagged *PCDH19* locus (magenta asterisk). **(B)** Immunocytochemistry (ICC) micrographs show PCDH19 localization using anti-HA and anti-FLAG antibodies in 2D WT neural rosettes. FLAG antibody gives more background staining than HA antibody; thus, HA antibody was used for the remainder of the study. DNA was stained with Bisbenzimide. Scale bar: 100 μm. **(C)** RT-qPCR confirmed the reduction of PCDH19 mRNA level in *PCDH19* KO hESCs. *p = 0.014; **p = 0.00174. Statistics were done with Dunn’s multiple comparisons test. Graphs are presented as mean ± standard error of the mean. **(D)** ICC micrographs show the loss of HA-FLAG tagged PCDH19 in *PCDH19* KO4-9 2D neural rosettes. DNA was stained with Bisbenzimide. Scale bar: 25 μm.

To examine PCDH19 expression and localization patterns in mosaic cortical organoids, we generated female H9 hESCs with either homozygous HA-FLAG-tagged *PCDH19* (WT) or homozygous *PCDH19* KO using CRISPR/CAS9 genome editing (Figure 1A). We first knocked in a 51 bp HA-FLAG sequence at the C-terminus of PCDH19 (before the stop codon) to obtain multiple HA-FLAG-tagged hESC lines (Figure 1A; Supplementary Figure S1A). We validated the presence of the HA-FLAG tag in neural rosettes that were differentiated from one homozygous HA-FLAG-tagged *PCDH19* hESC line (Figure 1B; Supplementary Figure S1C) and one heterozygous tagged line (Supplementary Figure S1D) by immunocytochemistry analyses. These two lines maintained proper pluripotent stem cell markers (Supplementary Figures S1A,B). We then generated two *PCDH19* KO hESC lines (KO4 and KO10) by using a sgRNA targeting the first exon of *PCDH19* (Figure 1A, red asterisk). Indel patterns and predicted frameshift outcomes are listed in Supplementary Table S2. Since both KO lines are compound heterozygotes, we performed single-cell cloning to further ensure that both lines are homogenous clones. Indels in exon 1 resulted in a frameshift and introduction of an early stop codon, leading to a decrease in PCDH19 mRNA levels by more than 90% in PCDH19 KO4 line and ~ 80% in KO10 line by RT-qPCR (Figure 1C). However, both lines had complete loss of PCDH19 protein confirmed by immunoblotting and immunostaining with anti-HA antibody (Figure 1D; Supplementary Figure S2). Two single-cell clones, KO4-7 and KO10-1, were used in the subsequent mixing experiments.

### 3.2 PCDH19 localization in human cortical organoids

**Figure 2:**
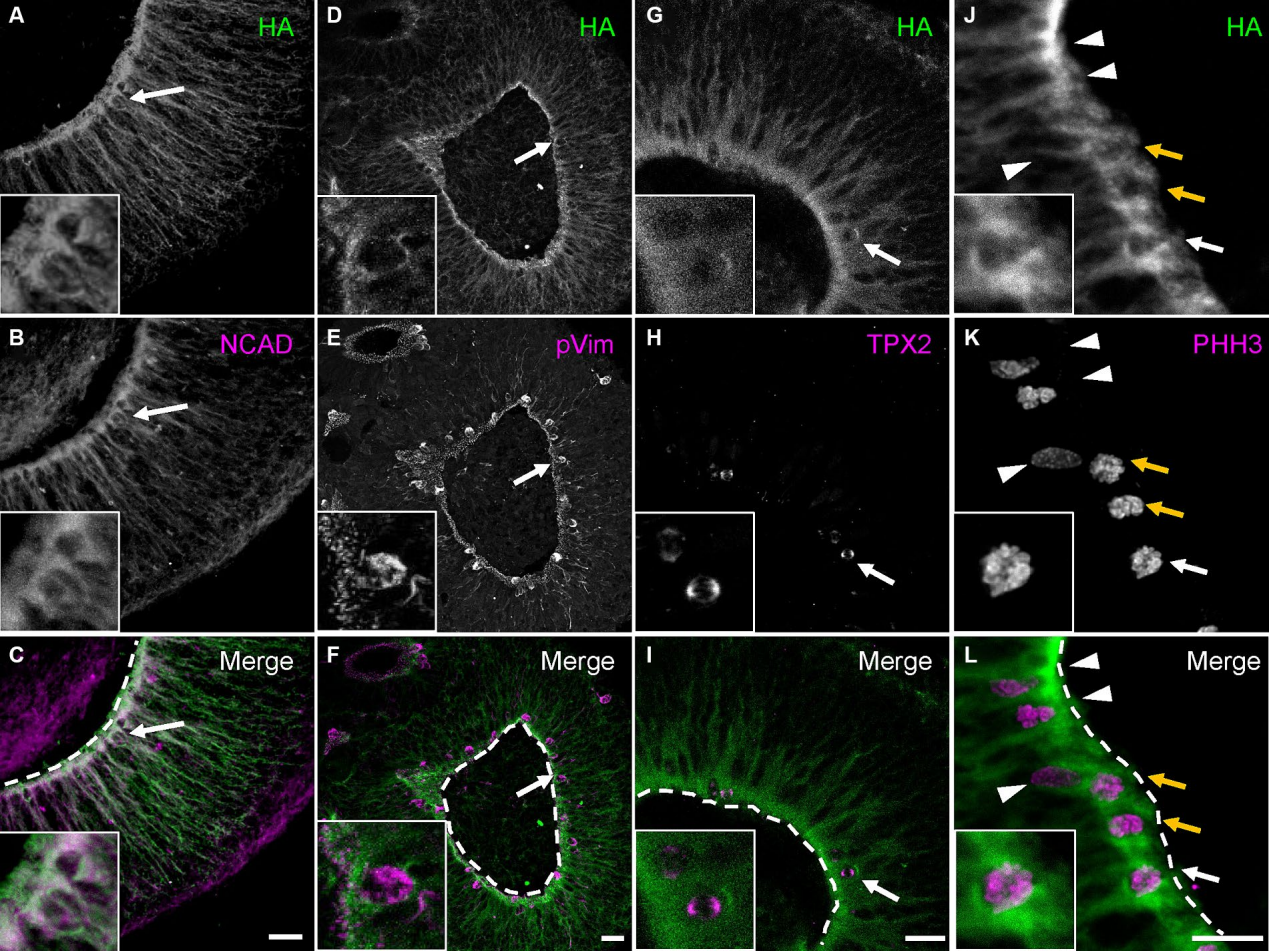
PCDH19 localizes predominantly to the apical surface and VZ/SVZ-like regions in day 20 hCOs. **(A–C)** Micrographs show PCDH19 co-localizes with NCAD near the apical lumen. The arrows denote an example of a double-labeled cell that is enlarged in the higher magnification insets at lower left. **(D–F)** PCDH19 localizes to the soma of dividing radial glia that were immunolabeled with anti-phosphorylated Vimentin antibody (arrows denote one example that is also shown in the higher magnification insets at lower left). **(G–I)** PCDH19 localized to the cell bodies of M-phase cells that were labeled by anti-TPX2 antibody (arrows; enlarged view in the insets at lower left). **(J–L)** PCDH19 labels cell bodies of G-M phase cells at the apical junction (arrows), but not the cells adjacent to the apical junction (arrow heads). G-M cells were labeled by anti-phosphohistone H3 antibody (PHH3). Anti-HA antibody was used to recognize the PCDH19 protein. Insets at lower left show a zoomed in view of the cell denoted by the single white arrow. White dash lines are apical junctions. VZ/SVZ, ventricular/subventricular zone. All scale bars are 25 μm.

To better understand PCDH19 function during early human corticogenesis, we differentiated WT hESC lines as 2D or 3D neural cultures using Dual-SMAD inhibition (Shi et al., 2012) or a single rosette brain organoid method we developed called self-organizing single rosette cortical organoids (SOSR-COs) (Takla et al., 2023; Tidball et al., 2023). PCDH19 mRNA levels and protein localization patterns were examined in the cultures. To determine the spatiotemporal expression pattern of PCDH19, we first analyzed PCDH19 mRNA levels throughout the 2D differentiation of a *PCDH19* WT (HA-FLAG tagged) line. We observed maximal PCDH19 transcript levels in 2D neural rosettes, while a voltage-gated sodium channel gene, *SCN1B*, exhibited the expected low expression in neural rosettes that subsequently increased with further differentiation to neurons (Supplementary Figure S3A). Similarly, we observed the highest PCDH19 transcript level in day 20 SOSR-COs at a time point of neural progenitor expansion in rosettes with limited neurogenesis (Supplementary Figure S3B; Tidball et al., 2023). This finding was confirmed by immunostaining with anti-HA antibody (Supplementary Figure S3C). In day 20 SOSR-COs, PCDH19, and NCAD co-localized to the apical adherent junctions and extended radially from the apical to basal surface (Figures 2A–C). Close examination of PCDH19 subcellular localization showed that it was found in the soma of dividing radial glia, identified by phosphorylated vimentin (pVIM) immunolabeling, in VZ-like regions (Figures 2D–F), and also localized to mitotic cells that expressed TPX2 (Figures 2G–I) or phosphohistone H3 (PHH3) (Figures 2J–M).

### 3.3 PCE hCO models show a cell sorting phenotype

**Figure 3:**
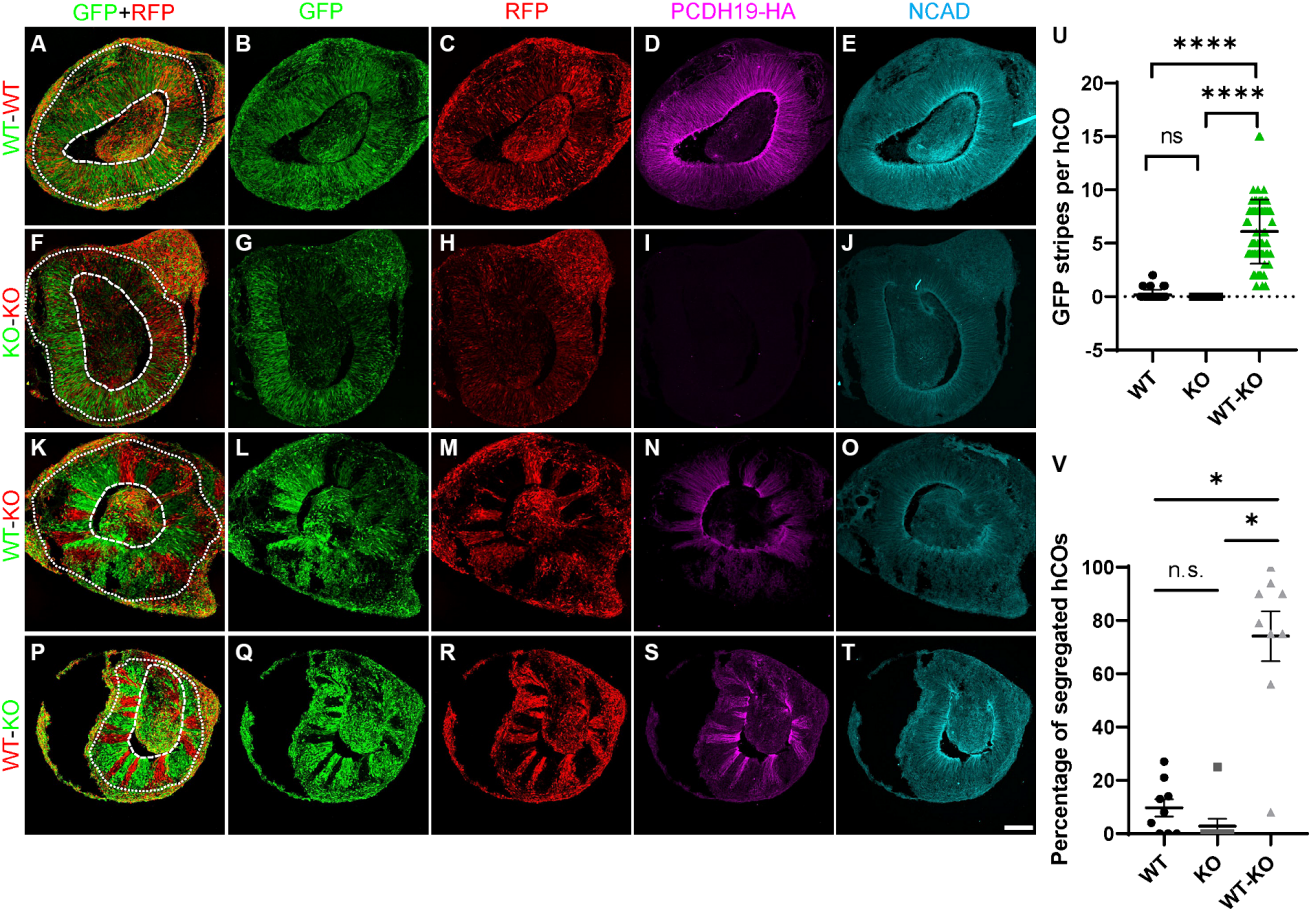
PCE hCOs show robust cell segregation phenotypes at day 20. **(A–E)** Day 20 hCOs derived from only WT cells do not show spatial segregation in VZ/SVZ-like regions **(A–C)** after 1:1 mixing of RFP- and GFP-labeled cells prior to hCO generation. The expected expression pattern of HA and NCadherin (NCAD) is seen **(D,E)**. **(F–J)** hCOs derived from only KO cells do not exhibit cell segregation in the VZ/SVZ-like regions **(F–H)**. KO cells stably expressing either GFP or RFP were mixed 1:1 prior to hCO formation. As expected, no HA is expressed **(I)** but NCAD expression is present **(J)**. **(K–O)** GFP-labeled *PCDH19* WT cells mixed 1:1 with RFP-labeled KO cells prior to hCO generation sorted into distinct GFP-only or RGP-only stripes in VZ/SVZ regions of day 20 PCE hCOs **(K–M)**. The cell segregation is also apparent in the HA-labeled image **(N)**. **(P–T)** A similar cell segregation phenotype was observed in day 20 PCE hCOs that were differentiated from a mixed hESC culture of WT-RFP/KO-GFP (1:1) cells **(P–R)**, and the pattern was also apparent in the HA-immunolabeled image **(S)**. PCDH19 expresses only in WT cells, not in KO cells **(D,I,N,S)**, while NCAD expresses in hCOs of all culture conditions **(E,J,O,T)**. Scale bar: 100 μm. The dashed circular lines mark the apical lumen, and the dotted circular lines denote the outer edge of the SVZ below the emerging cortical mantle. **(U)** Quantification of GFP stripes per hCO. The number of GFP stripes is significantly higher in PCE (WT-KO) hCOs than that in either WT only or KO only hCOs (****p < 0.0001). There is no significant difference between WT only and KO only cultures (^ns^*p* > 0.9999). Images were acquired on a Leica SP5 confocal imaging system. Four biological replicates were included in the plot. Graphs are presented as mean ± SEM. **(V)** Percentage of hCOs with striping (red) in each biological replicate. Significantly higher numbers of PCE hCOs displayed stripes compared to either WT or KO hCOs. *, *P*-value (PCE vs. WT) = 0.0184; *, *P*-value (PCE vs. KO) = 0.028; n.s., *P*-value (KO vs. WT) > 0.9999. *PCDH19* KO4-7 cells were used in these experiments. Numbers of biological replicates: 7 for the WT condition; 3 in the KO condition; and 9 in the PCE (WT-KO) condition. Statistical comparisons were made with Dunn’s multiple comparisons test. VZ/SVZ, ventricular zone/subventricular zone.

To model and compare the effects of mosaic PCDH19 expression *in vitro*, we used three mixed hESC line conditions to generate hCOs: pure WT, pure KO, and a WT/KO (PCE) mix (at a 1:1 ratio), which represent healthy control, carrier (hemizygous) male, and PCE patient (mosaic) conditions, respectively. We first established WT and KO cell lines that stably express either GFP or RFP reporters using lentivirus to examine cell segregation after mixing. Minimal to no cell segregation (striping) was seen in day 20 hCOs derived from pure WT or pure KO GFP/RFP mixes (Figures 3A–J,U,V). In contrast, we found robust separation of *PCDH19* KO and WT cells in VZ/SVZ areas of WT/KO mosaic hCOs at day 20 (Figures 3K–N, P–S,U,V). No differences were seen when GFP and RFP reporter labeling of WT and KO were switched in mosaic hCOs (Figures 3K,P). The average number of GFP+ or RFP+ stripes per organoid in WT/KO mixes was ~6, and over 70% of hCOs showed robust cell sorting (Figures 3U,V). Immunoblotting with anti-HA antibody confirmed the expected PCDH19 expression levels in the three hCO conditions at day 20 (Supplementary Figure S4F).

**Figure 4:**
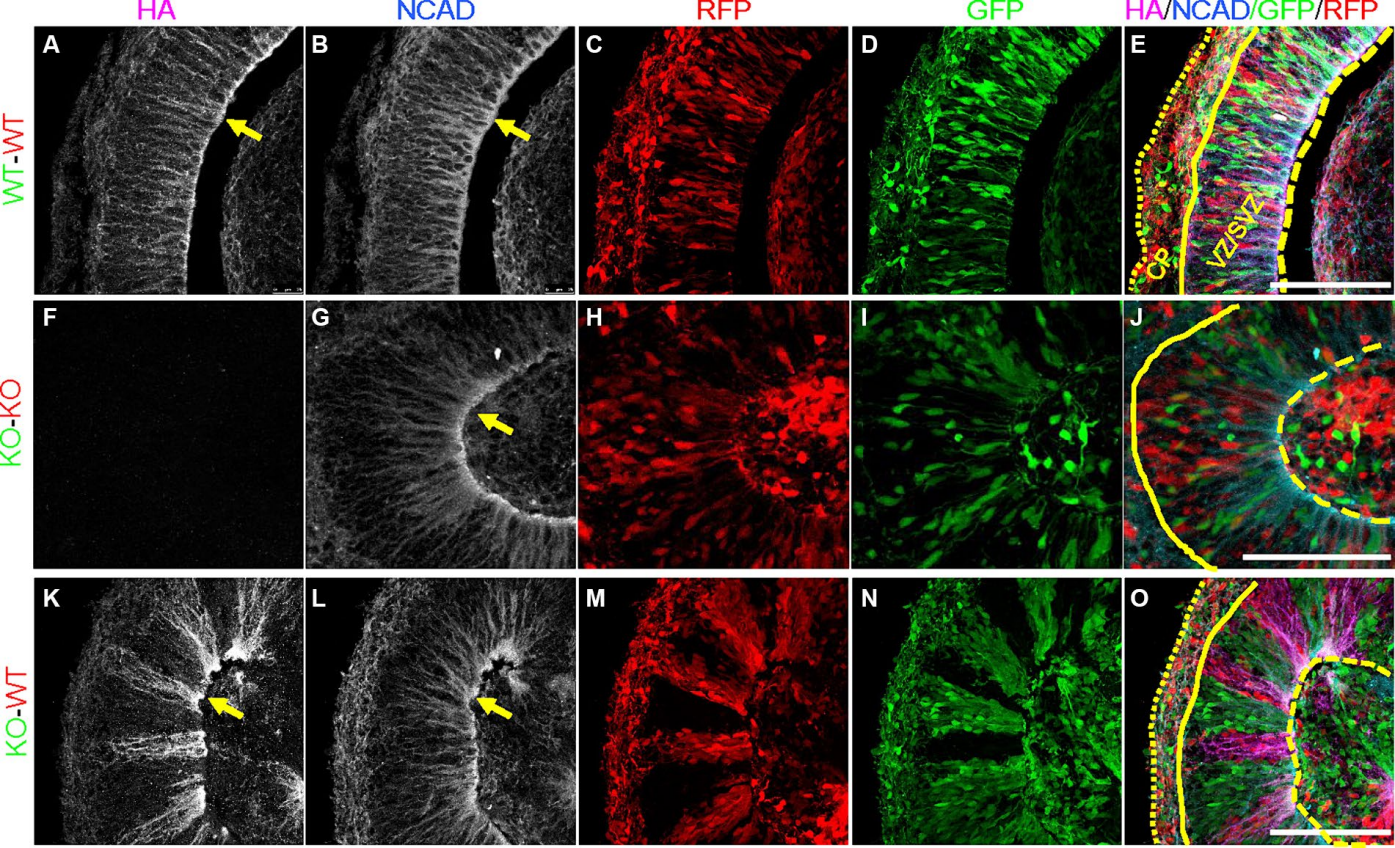
Altered expression of PCDH19-HA and NCAD in day 20 PCE hCOs. **(A–E)** NCAD and PCDH19-HA were fairly uniformly expressed in VZ/SVZ regions in the WT (*WT-GFP/WT-RFP = 1:1*) hCOs with highest expression at the apical lumen (yellow arrows). Cells in the VZ/SVZ region display typical radial glial cell morphology, with apical processes attached to the apical surface, and basal processes extending basally. **(F–J)** KO (*KO-GFP/KO-RFP = 1:1*) hCOs show grossly normal expression pattern of NCAD in the absence of PCDH19-HA, except for less distinct somal NCAD expression. **(K–O)** Crowded subcellular pattern of PCDH19-HA and NCAD in the VZ/SVZ area of PCE (*WT-RFP/KO-GFP = 1:1*) hCOs. PCDH19-HA expresses only in WT cells, while NCAD is expressed in both WT and KO cells. Both proteins show more irregular apical-basal processes. Cells in either WT or KO stripes appear to be crowed at the apical junction, and exhibit a more irregular radial glial cell morphology. *PCDH19* KO4-7 cells were used in the current experiment. VZ/SVZ, ventricular zone/subventricular zone. All scale bars are 100 μm. Yellow arrows show co-localization of PCDH19-HA and NCAD. Yellow dashed lines denote the apical lumen, the solid yellow line denotes the outer edge of the VZ/SVZ-like region, and the dotted yellow line is at the outer edge of the organoid above the emerging cortical plate.

**Figure 5:**
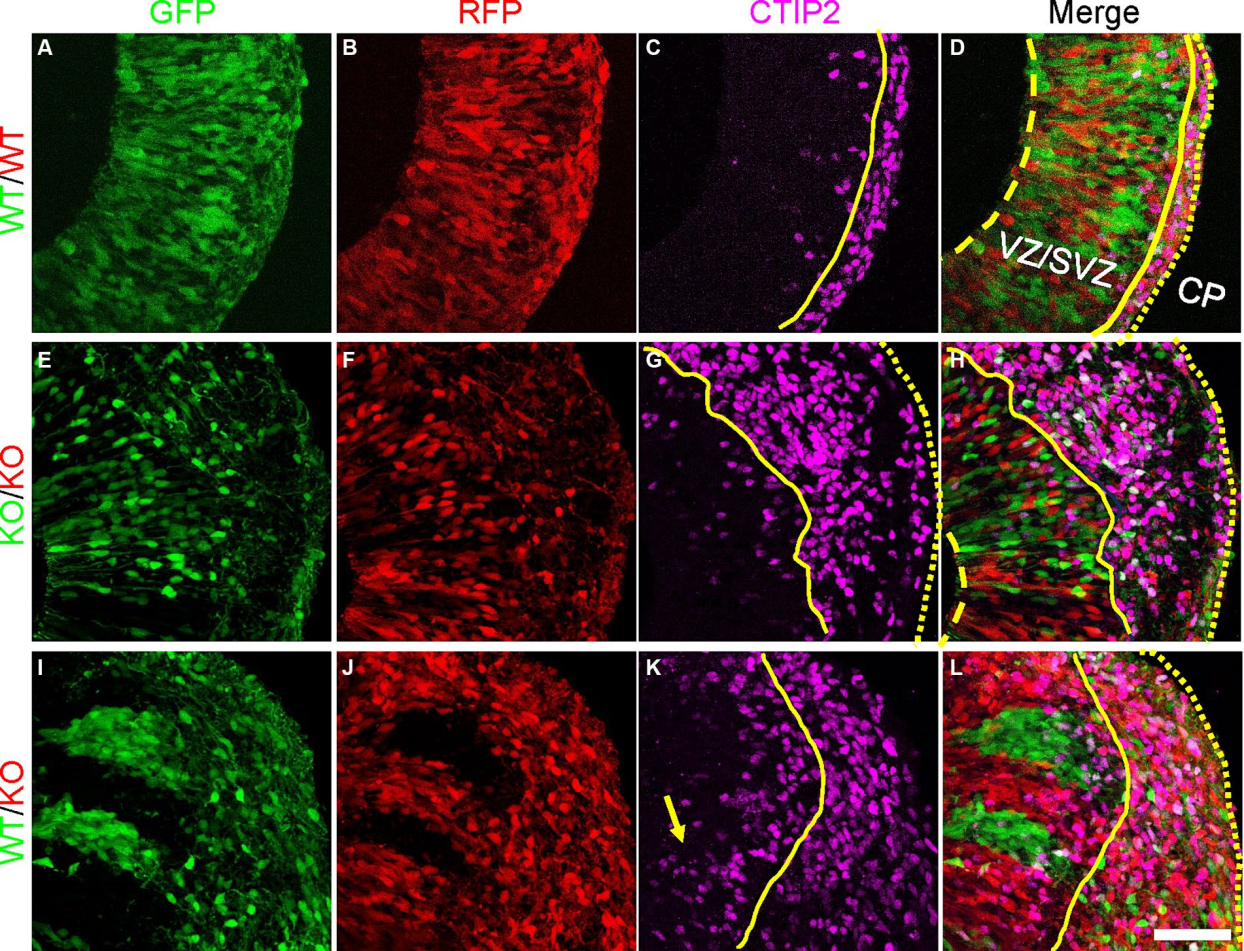
Atypical CTIP2 expression pattern in day 20 PCE hCOs. **(A–D)** WT (*WT-GFP/WT-RFP = 1:1*) only hCOs; **(E–H)** KO (*KO-GFP/KO-RFP = 1:1*) only hCOs; **(I–L)** PCE (*WT-GFP/KO-RFP = 1:1*) hCOs. *PCDH19* KO4-7 cells were used in the current experiment. Early born cortical neurons expressing CTIP2 were seen ectopically in VZ/SVZ regions of PCE hCOs (**K**, yellow arrow), while the expression of CTIP2 was largely restricted to the CP-like region in the WT hCOs **(C)**. An intermediate phenotype was seen in the KO only hCOs **(G)**. The dashed yellow lines label the apical lumen (not visible in **L**), the solid yellow lines are at the outer edge of the VZ/SVZ-like regions, and the dotted yellow line denotes the outer edge of the hCO, above the emerging CP. Scale bars are 100 μm. VZ/SVZ, ventricular zone/subventricular zone.

Live hCO imaging and confocal imaging of fixed hCO sections showed that no cell segregation (other than that expected from thin clonal cortical columns) arose from any culture condition before ~day 12 (Supplementary Figures S4A,E). In addition, *PCDH19* KO or mosaicism did not affect measures of early (day 9) hCO size or lumen formation (Supplementary Figures S4B–D). Furthermore, hCOs from all mixing conditions express the VZ radial glial stem cell markers, PAX6 and NESTIN (Supplementary Figure S5A). *PCDH19* KO cells proliferate in both KO only and in the mosaic condition (Supplementary Figure S5B). However, at or after ~day 14, cell sorting appeared nearly exclusively in WT/KO mosaic hCOs (Supplementary Videos 1E–H), and no substantial cell sorting arose in pure WT or pure KO conditions throughout the SOSR-CO differentiation (Figures 3–5; Supplementary Figures S6–S8; Supplementary Videos S1A–D). The cell sorting phenotype persisted in the day 35 organoids, but the phenotype was not robustly visible by 4–5 weeks when the rosette morphology became altered or they no longer contained a single main rosette (Supplementary Figure S7B). To model mild skewing of RXI that is likely to occur in patients, we next generated PCE organoids with different ratios of WT:KO cells (1:1, 2:1, 1:2) and found significant cell segregation from all mixing conditions in comparison to WT only and KO only cultures (Supplementary Figures S6, S8), with no quantitative difference in striping between the three mosaic conditions (Supplementary Figure S6B, adjusted *P* > 0.999).

### 3.4 Altered subcellular localization of PCDH19 and NCAD in day 20 PCE hCOs

In order to validate that our PCE organoid model contains mosaic expression of PCDH19, we immunostained day 20 WT, KO and WT/KO (PCE) hCOs with anti-HA antibody to identify PCDH19 protein, as well as N-cadherin (NCAD), a PCDH19 interacting protein (Cooper et al., 2016; Hoshina et al., 2021; Hudson et al., 2021). We confirmed that PCDH19 localizes to the apical junction in all WT organoids, and displays distinct apical-basal patterns (Figures 3D, 4A). PCDH19-HA tagged protein was not detected in any KO organoids that we examined (Figures 3I, 4F). In WT/KO mosaic organoids, PCDH19 was only observed in WT cells as expected (Figures 3K–N, P–S), while NCAD was present in cells of both genotypes (Figures 3E,J,O,T). Higher magnification views of fixed hCO cryosections, as well as co-labeling with the radial glial stem cell marker PAX6 and deep layer neuronal marker CTIP2 revealed that cell sorting in WT/KO hCOs occurs only in the VZ/SVZ regions and not in the emerging CP-like region (Figures 4M–O; Supplementary Figures S9K–O). In pure WT or pure KO hCOs, cells in the VZ/SVZ regions exhibited a typical radial glial-like morphology (Figures 4A–D,G), and both PCDH19 and NCAD were uniformly localized to the apical junction and cell–cell junctions in VZ/SVZ regions (Figures 4A,B,G). In WT/KO mosaic hCOs, in contrast, radial glial morphologies in the VZ/SVZ were somewhat altered in both WT and KO stripes (Figures 4K–N), showing a “crowded” pattern of PCDH19 and NCAD within each stripe with clumping at the apical surface (Figures 4K,L). Similar cellular phenotypes were also present when WT and KO cells were mixed in different ratios (Supplementary Figure S8). NCAD expression in WT/KO mosaic hCOs also appeared to have a more variable apical to basal distance and more disorganized radial processes compared to all WT or all KO hCOs (Figures 4B,G,L). Moreover, PCDH19-HA expression occasionally appeared much higher in WT cells of mosaic hCOs compared to pure WT hCOs (compare Figure 4A with Figure 4K), although this finding was variable between organoid batches.

### 3.5 Abnormal early cortical lamination in PCE brain organoids

To assess whether the radial glia with altered morphology and changes in PCDH19 and NCAD expression impact early cortical lamination, we immunostained day 20 hCO cryosections for the deep layer (early born) cortical neuron marker CTIP2 (BCL11B). In pure WT or pure KO hCOs, CTIP2 primarily localized to the CP-like region, with few labeled cells in the VZ/SVZ region (Figures 5A–H). In WT/KO mosaic hCOs, in contrast, larger numbers of CTIP2+ neurons remained in the VZ/SVZ regions (Figures 5I–L). We quantified mean intensity of CTIP2 in either VZ/SVZ or CP region in all three mixing conditions. We found a non-statistically significant trend for WT/KO having a higher ratio of CTIP mean intensity in VZ/CP regions vs. CP regions (Supplementary Figure S10A, adjusted *p* = 0.2629). In addition, we found significantly increased CTIP2+ cell numbers in KO stripes compared to those in WT stripes (Supplementary Figure S10B, adjusted *p* = 0.0286).

## 4 Discussion

Mosaicism due to the coexistence of *PCDH19*-WT and *PCDH19*-mutant cells from RXI in females is thought to impair cell–cell interactions leading to altered brain development. This idea is known as the “cellular interference” model and represents a leading hypothesis for why PCE exclusively affects heterozygous females and males with somatic mosaicism, but not hemizygous males (Dibbens et al., 2008). As a result of cellular interference, *PCDH19* WT cells and *PCDH19* mutant cells sort into distinct regions, but when and how these cell-sorting events occur during human cortical development is unknown. This spatial segregation has also been reported in the optic tectum of zebrafish (Cooper et al., 2015) and in the cortex of heterozygous *PCDH19* KO mice (Pederick et al., 2018). However, this “cellular interference” hypothesis has not been shown in human PSC derived models.

Unexpected X chromosome skewing during reprogramming and culture of female iPSC lines diminishes the ability to model RXI, which adds to the complexity of modeling PCDH19 mosaic expression *in vitro* (Pomp et al., 2011; Tomoda et al., 2012). Moreover, defining the percentage of cells that express the mutant vs. WT allele in any given experiment is challenging and labor intensive. To overcome these issues, we used CRISPR gene editing to first tag both *PCDH19* alleles with HA-FLAG and then to delete both tagged alleles in female hESCs. The KO lines show complete loss of PCDH19 protein. Mixing these KO cells with isogenic *PCDH19* WT hESCs at a 1:1 ratio mimics the mosaic PCDH19 expression that occurs with heterozygous female PCE patients *in vivo* due to RXI. As predicted, when the mixed KO and WT cells are used to generate hCOs they display a robust cell sorting phenotype that is not present in pure WT or pure KO hCOs. The cell sorting is restricted to VZ/SVZ regions in the hCO model, largely consistent with findings in heterozygous *PCDH19* KO mice, although the mice tend to have some incomplete segregation in the cortical plate and later cortex (Pederick et al., 2018). This difference may reflect the fact that radial glia process extension to the pial surface is not well maintained as hCOs grow. The more robust cell sorting pattern in the progenitor niche also likely reflects the higher expression of PCDH19 in the VZ/SVZ region (Figure 2; Supplementary Figure S3C). Thus, we speculate that PCDH19 is important to regulate radial glia cell expansion and/or polarity in VZ/SVZ region. To our knowledge, this is the first hPSC model that displays robust and consistent cell sorting phenotypes.

We found that cell sorting occurs in VZ/SVZ regions of early stage PCE hCOs. VZ/SVZ regions at this stage are comprised primarily of neural stem and progenitor cells (Tidball et al., 2023). The stem cells in the developing nervous system are radial glia characterized by long radial processes that facilitate the radial migration of newborn neurons from the VZ to the cortical mantle region. In WT hCOs, we found that PCDH19 expression is negligible in hESCs, highest at the neural rosette stage and then decreases to intermediate levels during subsequent neuronal differentiation, which is similar to a previous study of iPSC-derived 2D neural rosettes (Homan et al., 2018). As expected, we observed co-localization of PCDH19 with NCAD at apical junctions of neural rosettes, but we also found that PCDH19 is expressed in the soma of dividing radial glia present near the apical lumen of the VZ-like region of hCOs. The PCDH19 expression pattern and the cell sorting phenotype in VZ/SVZ regions of early PCE hCOs suggest that PCDH19 may play critical roles in early human neurodevelopment through regulating radial glial cell development during the formation or expansion of neural rosettes.

We also explored the influence of mosaic PCDH19 expression on early neurogenesis in hCOs. Cortical neurons produced in the VZ migrate radially to reach the cortical mantle. We found a trend for relatively more newly born CTIP2+ (deep cortical layer) neurons in the VZ/SVZ regions of WT/KO hCOs than in either pure WT or pure KO cultures. This aberrant neurogenesis may reflect premature differentiation of CTIP2+ neurons in PCE, or instead may result from altered neuronal migration. Premature (or accelerated) neurogenesis was previously found in 2D cortical neuron differentiations that were either from patient iPSCs that did not express PCDH19 (Homan et al., 2018; Alaverdian et al., 2023), or from mixed control and *PCDH19* mutant iPSC cultures (Borghi et al., 2021). The potential for altered neuronal migration is supported by prior studies showing altered *in vivo* or *in vitro* migration in PCE models or after *PCDH19* knockdown (Cooper et al., 2015; Pederick et al., 2016; Bassani et al., 2018; Pederick et al., 2018). Notably, the radial migration of the pyramidal neurons born in the VZ and SVZ area is facilitated by the basal processes of radial glia (Rakic, 1995a). Cells in VZ/SVZ regions of WT/KO mosaic hCOs not only segregated into distinct columns, but also appeared to be crowded in each column, exhibiting more indistinct elongated basal or apical processes, which likely compromised their ability to serve as a scaffold for the migrating neurons. The altered radial processes were occasionally and to a lesser extent observed in pure KO hCOs.

**Figure 6:**
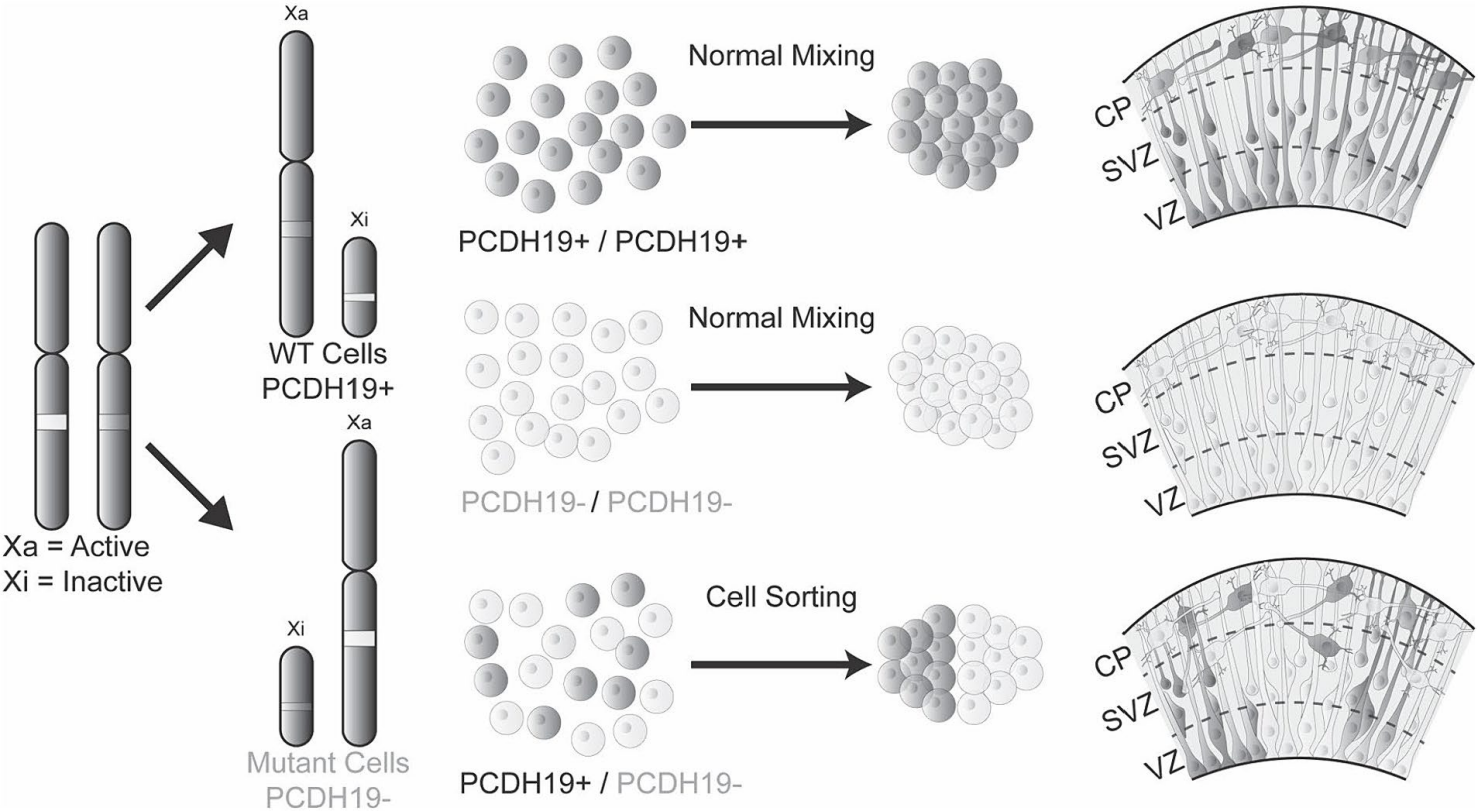
The mosaic hCO model of PCE reveals abnormal cell sorting and laminar organization during early cortical development. Healthy individuals or hemizygous male carriers have either WT *PCDH19* only or mutant P*CDH19* only cells, leading to homogenous cell–cell interactions that result in normal cell sorting in the VZ/SVZ region during early cortical development. However, in PCE individuals, mosaic expression of PCDH19 due to random X-inactivation or mosaic somatic mutations impair cell interactions between those expressing only WT *PCDH19* (PCDH19+) and those expressing only mutant *PCDH19* (PCDH19-), leading to sorting of two cell populations to distinct regions during early cortical development. The mosaic expression of PCDH19 and cell sorting can be modeled *in vitro* by mixing *PCDH19* WT hESCs and *PCDH19* KO hESC at different ratios followed by hCO differentiation. During early PCE hCO differentiation, PCDH19 and NCAD localization patterns are altered (not shown), and radial glia (dark gray and/or light gray cells) are disorganized in VZ/SVZ regions, which result in disorganization of early-born neurons in the emerging cortical plate.

Interestingly, the altered radial glia morphology in mosaic hCOs corresponded with changes in the expression patterns of PCDH19 and NCAD. In pure WT hCOs, both PCDH19 and NCAD show a distinct apical to basal pattern that is evenly distributed across the VZ/SVZ area of hCOs. However mosaic PCE hCOs show less distinct and likely more intense or dense expression PCDH19 and to a lesser extent NCAD. PCDH19 is a CAM that forms cis and trans binding with itself or other adhesion proteins such as NCAD (Biswas et al., 2010; Emond et al., 2011; Hoshina et al., 2021). We speculate that mosaic expression of PCDH19 may alter the radial glia cell division through the interaction with NCAD, as the NCAD/β-catenin signaling pathway has been previously implicated in the expansion defects of radial glia cells in Miller-Dieker Syndrome (MDS) (Iefremova et al., 2017) and later synaptic development in Pcdh19 heterozygous mice (Hoshina et al., 2021). In addition, NCAD expression regulates the differentiation and delamination of new neurons that arise from the radial glia (Zhang et al., 2010; Hatakeyama et al., 2014; Kawaguchi, 2020), and alterations in NCAD expression or function could lead to premature differentiation/delamination. Our results suggest that PCDH19 and NCAD together are important for guiding the proper cell–cell sorting of radial glia. The presence of two cell populations, pure WT and pure *PCDH19* KO cells, in the VZ/SVZ area likely affects proper cell–cell recognition or interaction leading to spatial segregation, which in turn affects the development of radial glial processes that then likely alters the migration of newly born neurons (Figure 6). However, whether and how cell segregation impacts eventual neuronal network function remains to be determined.

The current study has several limitations. Although the cell segregation phenotype is extremely robust, the number and size of cell-segregated columns in VZ/SVZ areas varies between hCOs within the same batch, despite using the same mixing ratio of WT and KO cells. This variability poses hurdles to probe the exact underlying cellular mechanisms and potential outcomes of cell sorting and underscores the importance of using multiple cells lines and multiple biological replicates. We have recently reported the effects of mixing male P*CDH19* CRISPR KO and isogenic control cells (modeling mosaic males) to demonstrate the utility of our single-rosette hCOs, SOSR-COs (also used here) for revealing cell segregation phenotypes compared to multirosette hCO protocols (Tidball et al., 2023). Future studies combining the use of male carrier iPSCs carrying loss-of-function variants in *PCDH19* and variant-corrected cells, or mixing female patient iPSCs that only express WT or mutant *PCDH19* will be useful to further illuminate the underlying causes of cell sorting in PCE hCO models. Our work with the female hESC PCE model explored only relatively early (3-week) hCOs and future investigations of the role PCDH19 mosaicism plays in altering cortical development and function using later (4-5-month) organoids are necessary. Another limitation is that this study focused only on dorsally patterned hCOs that give rise to excitatory neurons. Exploring potential effects of PCDH19 mosaicism on ganglionic eminence stem cell niches and interneuron development is an important future direction. Finally, it remains to be determined whether early cell segregation of neural stem cells in VZ/SVZ regions leads to critical disease phenotypes such as seizures and intellectual disability, or whether these arise independently from later changes in synaptogenesis or other neurodevelopmental processes. Nonetheless, our PCE hCO model offers a platform for mechanistic studies of how *PCDH19* mosaicism leads to PCE phenotypes, and for testing precision therapies for this severe DEE.

## Data availability statement

The original contributions presented in the study are included in the article/Supplementary material, further inquiries can be directed to the corresponding author.

## Ethics statement

The studies involving human embryonic stem cells were approved by the University of Michigan Human Pluripotent Stem Cell Research Oversight (HPSCRO) Committee. The studies were conducted in accordance with the local legislation and institutional requirements. The human samples used in this study were acquired from H9 human embryonic stem cell line obtained from a non-profit organization, WiCell Research Institute. Written informed consent for participation was not required from the participants or the participants’ legal guardians/next of kin in accordance with the national legislation and institutional requirements.

## Author contributions

WN: Conceptualization, Formal analysis, Funding acquisition, Investigation, Visualization, Writing – original draft, Writing – review & editing. LD: Data curation, Formal analysis, Investigation, Writing – review & editing. SM-P: Formal analysis, Investigation, Writing – review & editing. AT: Formal analysis, Writing – review & editing. RS: Formal analysis, Writing – review & editing. KS: Investigation, Validation, Visualization, Writing – review & editing. JP: Conceptualization, Funding acquisition, Project administration, Resources, Supervision, Writing – review & editing.

## Footnotes

^1^ https://genome.ucsc.edu/
