# Supplementary material for: Abnormal cell sorting and altered early neurogenesis in a human cortical organoid model of Protocadherin-19 clustering epilepsy
Source: Front Cell Neurosci. 2024 Apr 4;18:1339345. doi: 10.3389/fncel.2024.1339345 (PMC11024992; doi:10.3389/fncel.2024.1339345)
Supplement: Supplementary file 1 [file Table_1.docx]

**Supplementary Table 1: List of antibodies used in immunocytochemistry and immunoblotting analyses.**

| **Name** | **Species** | **Dilution** | **Vendor** | **Catalog #** |
| --- | --- | --- | --- | --- |
| **Immunocytochemistry primary antibody** | | | | |
| CTIP2 | Rat | 1:300 | Abcam | ab18465 |
| Histone H3 (S28) | Rat | 1:5000 | Abcam | ab10543 |
| PAX6 | Rabbit | 1:1000 | MBL Int. | PD022 |
| SOX2 | Rabbit | 1:800 | Millipore | ab5603 |
| NESTIN | Mouse | 1:300 | Millipore | MAB5326 |
| MKI67 | Rabbit | 1:1000 | ThermoFisher | MA5-14520 |
| SSEA4 | Mouse | 1:125 | Abcam | ab16287 |
| PCDH19 | Mouse | 1:100 | Abcam | ab57510 (discontinued) |
| NCAD | Mouse | 1:500 | Life technologies | 33-3900 |
| HA | Rabbit | 1:250 | Cell signaling | 3724S |
| HA | Mouse | 1:100 | Cell signaling | 2367S |
| Phospho-Vimentin (pVim) | Mouse | 1:1000 | MBL Int | D076-3 |
| TPX2 | Rabbit | 1:500 | Novus Biologicals | NB500-179 |
| FLAG | Mouse | 1:250 | Sigma | F1804 |
| **Immunocytochemistry secondary antibody** | | | | |
| anti-Rabbit Alexa Fluor 488 | Goat | 1:400 | ThermoFisher | A-11034 |
| anti-Mouse Alexa Fluor 568 | Goat | 1:400 | ThermoFisher | A-11031 |
| anti-Rabbit Alexa Fluor 568 | Goat | 1:400 | ThermoFisher | A-11036 |
| anti-Rat Alexa Fluor 647 | Goat | 1:400 | ThermoFisher | A-21247 |
| **Immunoblotting blotting primary antibody** | | | | |
| GAPDH |  | 1:1000 | Abcam | AB9484 |
| ACTIN | Mouse | 1:500 | Santa Cruz | sc-8432 |
| **Immunoblotting secondary antibody** | | | | |
| Anti-Mouse IgG (H + L)-HRP Conjugate | Goat | 1:2000 | Biorad | 1706516 |
| Anti-Rabbit IgG (H + L)-HRP Conjugate | Goat | 1:2000 | Biorad | 1706515 |
