# Supplementary material for: Abnormal cell sorting and altered early neurogenesis in a human cortical organoid model of Protocadherin-19 clustering epilepsy
Source: Front Cell Neurosci. 2024 Apr 4;18:1339345. doi: 10.3389/fncel.2024.1339345 (PMC11024992; doi:10.3389/fncel.2024.1339345)
Supplement: Supplementary file 3 [file Presentation_1.PPTX]

## Slide 1
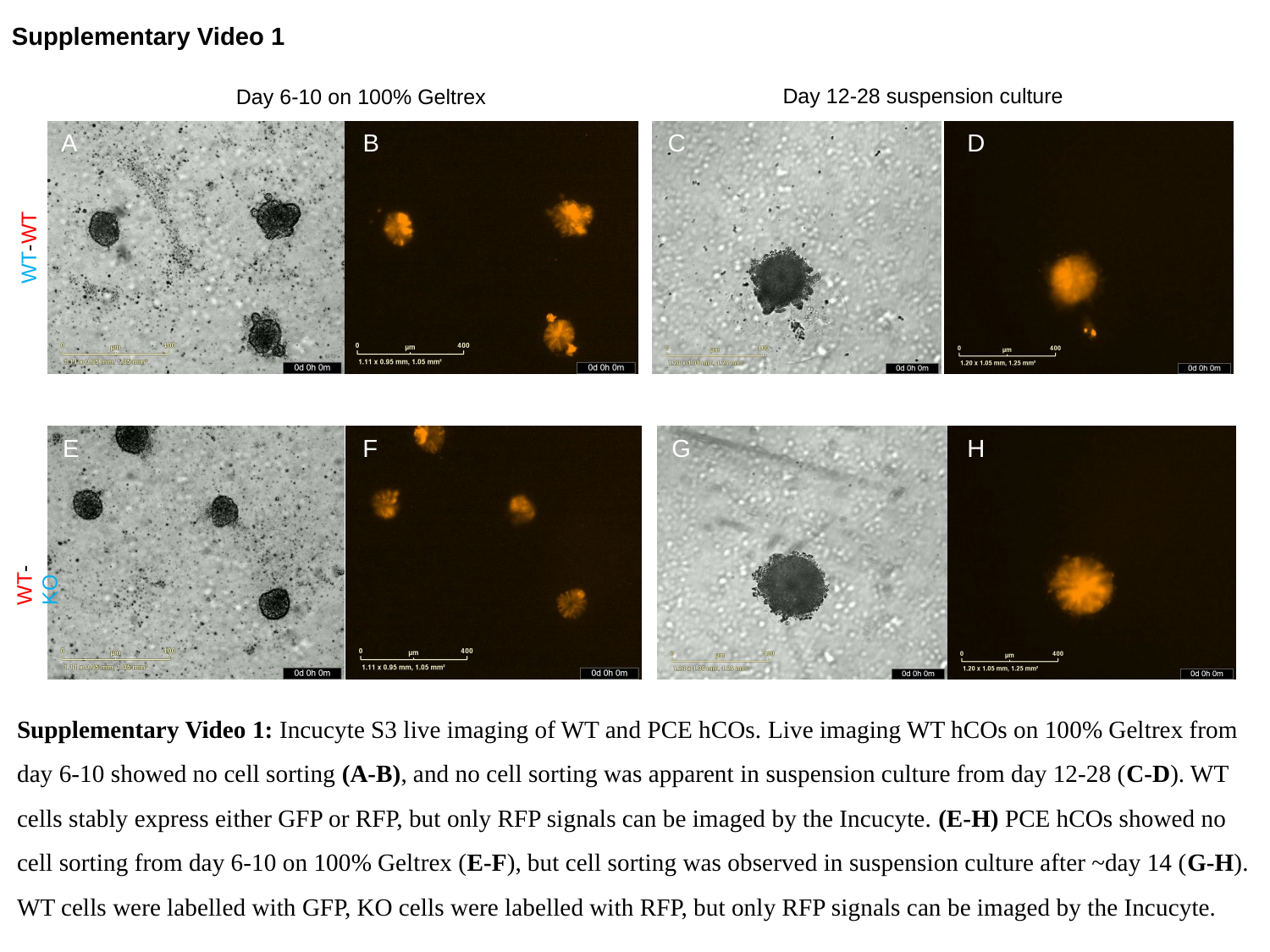

Supplementary Video 1
Day 12-28 suspension culture
Day 6-10 on 100% Geltrex
A
B
C
D
WT-WT
E
F
G
H
WT-KO
Supplementary Video 1: Incucyte S3 live imaging of WT and PCE hCOs. Live imaging WT hCOs on 100% Geltrex from day 6-10 showed no cell sorting (A-B), and no cell sorting was apparent in suspension culture from day 12-28 (C-D). WT cells stably express either GFP or RFP, but only RFP signals can be imaged by the Incucyte. (E-H) PCE hCOs showed no cell sorting from day 6-10 on 100% Geltrex (E-F), but cell sorting was observed in suspension culture after ~day 14 (G-H). WT cells were labelled with GFP, KO cells were labelled with RFP, but only RFP signals can be imaged by the Incucyte.
