# Supplementary material for: Abnormal cell sorting and altered early neurogenesis in a human cortical organoid model of Protocadherin-19 clustering epilepsy
Source: Front Cell Neurosci. 2024 Apr 4;18:1339345. doi: 10.3389/fncel.2024.1339345 (PMC11024992; doi:10.3389/fncel.2024.1339345)
Supplement: Supplementary file 4 [file Data_Sheet_1.docx]

**Supplementary Materials**

**Supplementary Figure 1**: Confirmation of *PCDH19* HA-FLAG tagged and KO hESC lines. **(A)** RT-qPCR confirmed mRNA expression levels of PCDH19 and the pluripotency marker NANOG in multiple *PCDH19* HA-FLAG tagged hESC cell lines. ACTB encodes b-actin. 1B4 and 1G1 are homozygous HA-FLAG tagged lines. 2E6 and 1B8 are heterozygous HA-FLAG tagged lines. 1G1 was used as the WT line in this study. **(B)** Immunocytochemistry analyses confirmed that three *PCDH19* HA-FLAG tagged hESC lines (1B4, 1G1, and 2E6) maintain the pluripotent stem cell markers SOX2 and SSEA4. Scale bar: 100µm. (**C-D**) Immunostaining using both anti-HA and anti-PCDH19 antibodies showed similar localization patterns of PCDH19 in hCOs that were derived from HA-FLAG tagged hESC cell line 1G1 (**C**) and 2E6 (**D**). Scale bar: 25 µm. DNA was stained with Bisbenzimide.

**Supplementary Figure 2:** Confirmation of PCDH19 knockout using immunoblotting and immunostaining. Immunoblotting using anti-HA antibody confirmed the reduction of PCDH19-HA protein levels in *PCDH19* KO hESC cells (**A**) and 2D neural rosettes (**B**). WT is HA-FLAG tagged 1G1 clone, KO4-7 and KO4-9 have the same indel patterns after gene editing, and KO10-1 and KO10-3 have the same indel patterns. **(C)** ICC micrograph shows PCDH19-HA knockout in 2D neural rosettes using anti-HA antibody. DNA was stained with Bisbenzimide. Scale bar is 25 µm.

**Supplementary Figure 3:** Spatiotemporal expression patterns of PCDH19 during hCO differentiation of *PCDH19-*HA-FLAG tagged hESCs. **(A)** RT-qPCR shows highest PCDH19 transcript levels at the 2D neural rosette stage and lower expression in neurons. In contrast, *SCN1B*, the beta-1 subunit of voltage-gated sodium channels, showed higher expression levels in neurons. ACTB is an internal control. **(B)** RT-qPCR showing highest PCDH19 transcript levels in day 20 hCOs that consist of primarily neural rosettes. GAPDH is an internal control. Day 0 represents stem cell stage. **(C)** The flow chart on top shows the differentiation process of hCOs. Immunocytochemistry analyses using anti-HA antibody show robust and distinct expression pattern of PCDH19-HA at the apical junctions of day 20 hCOs. Scale bars: 100 µm.

**Supplementary Figure 4:** No cell sorting was observed in early-stage PCE hCOs between days 6-12. **(A)** Confocal images of cryosections of day 9 PCE GFP-RFP (1:1 mix) hCOs confirmed no cell sorting phenotype. *PCDH19* KO10 cells were used in this mixing experiment. Scale bar: 100µm. **(B-D)** Quantification of structural measurements including organoid size, lumen area/hCO area, and lumen circularity show no obvious structural defects in day 6 hCOs. Images were taken with EVOS live imaging system. P-value >0.999 (Kruskal-Wallis test with multiple comparison). Three biological replicates were included in the assay. Graphs are presented as mean ± SEM. **(E)** Confocal images of cryosections of day 12 PCE hCOs (mixture as in **A**) confirmed no cell sorting phenotype. *PCDH19* KO4 cells were used in this experiment. Scale bar: 100µm. **(F)** Immunoblotting using anti-HA antibody confirmed PCDH19-HA knockdown in day 20 hCOs.

**Supplementary Figure 5.** Early-stage hCOs express radial glial cell markers. Whole mounts of day 12 hCOs were co-immunostained with PAX6 and NESTIN **(A)**, and MKI67 **(B)** antibodies. DNA was stained with Bisbenzimide. *PCDH19* KO7-4 cells were used. WT-GFP and KO-RFP are mixed at ratio 1:1. All scale bars are 100 µm.

**Supplementary Figure 6.** hCOs differentiated from different mixes of WT and KO cells show cell sorting phenotype. **(A)** hCOs were generated with different ratios of WT-KO cells (1:1, 2:1, 1:2), as well as with WT only and KO only cells. *PCDH19* KO-10 cells were used in this mixing experiment. **(B)** Quantification of cell segregation phenotype from all mixes. 1:1, 2:1, and 1:2 ratios showed significant cell segregation compared to WT and KO cultures (****, P-value < 0.0001, Kruskal-Wallis test with multiple comparison), but there is no significant difference between 1:1 and 2:1 (ns, P-value > 0.999, Kruskal-Wallis test with multiple comparison). Three biological replicates were included in the plot. Graphs are presented as mean ± SEM. All scale bars are 200 µm.

**Supplementary Figure 7.** Reduction of cell sorting phenotype in later stage PCE hCOs. Cell sorting phenotype in day 35 PCE hCOs (**B**) is not as robustly visible as it is in day 20 organoids (**A**). Yellow arrows point to subtle cell segregation areas at 35 days. All scale bars are 250 µm.

**Supplementary Figure 8.** Altered subcellular localization of PCDH19 and NCAD in PCE hCOs with different mixing ratios of WT and KO cells. *PCDH19* KO-10 cells were used in this mixing experiment. Cryosections of hCOs were co-immunostained with anti-HA and -NCAD antibodies. DNA was stained with Bisbenzimide. **(A-D)** WT-GFP/WT-RFP ratio 1:1; **(E-H)** KO-GFP only; **(I-L)** WT-GFP/WT-RFP ratio 1:1; **(M-P)** WT-GFP/WT-RFP ratio 2:1; **(Q-T)** WT-GFP/WT-RFP ratio 1:2. All scale bars are 100 µm.

**Supplementary Figure 9.** PAX6 and CTIP2 expression patterns in day 20 PCE hCOs. Cryosections of hCOs were co-immunostained with anti-PAX6 (C, H, M) and -CTIP2 (D, I, N) antibodies. DNA was stained with Bisbenzimide. *PCDH19* KO10-1 cells were used. **(A-E)** WT-GFP/WT-RFP ratio 1:1; **(F-J)** KO-GFP/KO-RFP ratio 1:1; **(K-O)** WT-GFP/KO-RFP ratio 1:1. All scale bars are 50µm.

**Supplementary Figure 10.** Quantification of CTIP2 intensity in day 20 PCE hCOs. **(A)** The ratio of CTIP mean intensity in VZ/SVZ regions vs. CP regions was elevated in mosaic KO-RFP/WT-GFP hCOs, compared to KO hCOs and WT hCOs. P values = 0.2629 (Kruskal-Wallis test with multiple comparison). **(B)** In mosaic KO-RFP/WT-GFP hCOs, more CTIP2+ cells co-express RFP than GFP, suggesting premature neurogenesis in KO cells. P values = 0.0286 (Mann Whitney test).

**Supplementary Video 1:** Incucyte S3 live imaging of WT and PCE hCOs. Live imaging of WT hCOs on 100% Geltrex from days 6-10 showed no cell sorting **(A-B)**, and no cell sorting was apparent in suspension culture from day 12-28 (**C-D**). WT cells stably express either GFP or RFP, and only RFP signals can be imaged by the Incucyte. **(E-H)** PCE hCOs showed no cell sorting from days 6-10 on 100% Geltrex (**E-F**), but cell sorting was observed in suspension culture after ~day 14 (**G-H**). WT cells were labeled with GFP, *PCDH19* KO4 cells were labeled with RFP, and only RFP signals can be imaged by the Incucyte.

**Supplementary Table 1:** List of antibodies used in immunocytochemistry and immunoblotting analyses.

**Supplementary Table 2:** Indel patterns and predicted frameshift outcomes in *PCDH19* KO lines.
