# Supplementary material for: Abnormal cell sorting and altered early neurogenesis in a human cortical organoid model of Protocadherin-19 clustering epilepsy
Source: Front Cell Neurosci. 2024 Apr 4;18:1339345. doi: 10.3389/fncel.2024.1339345 (PMC11024992; doi:10.3389/fncel.2024.1339345)

Supplementary Figure 1

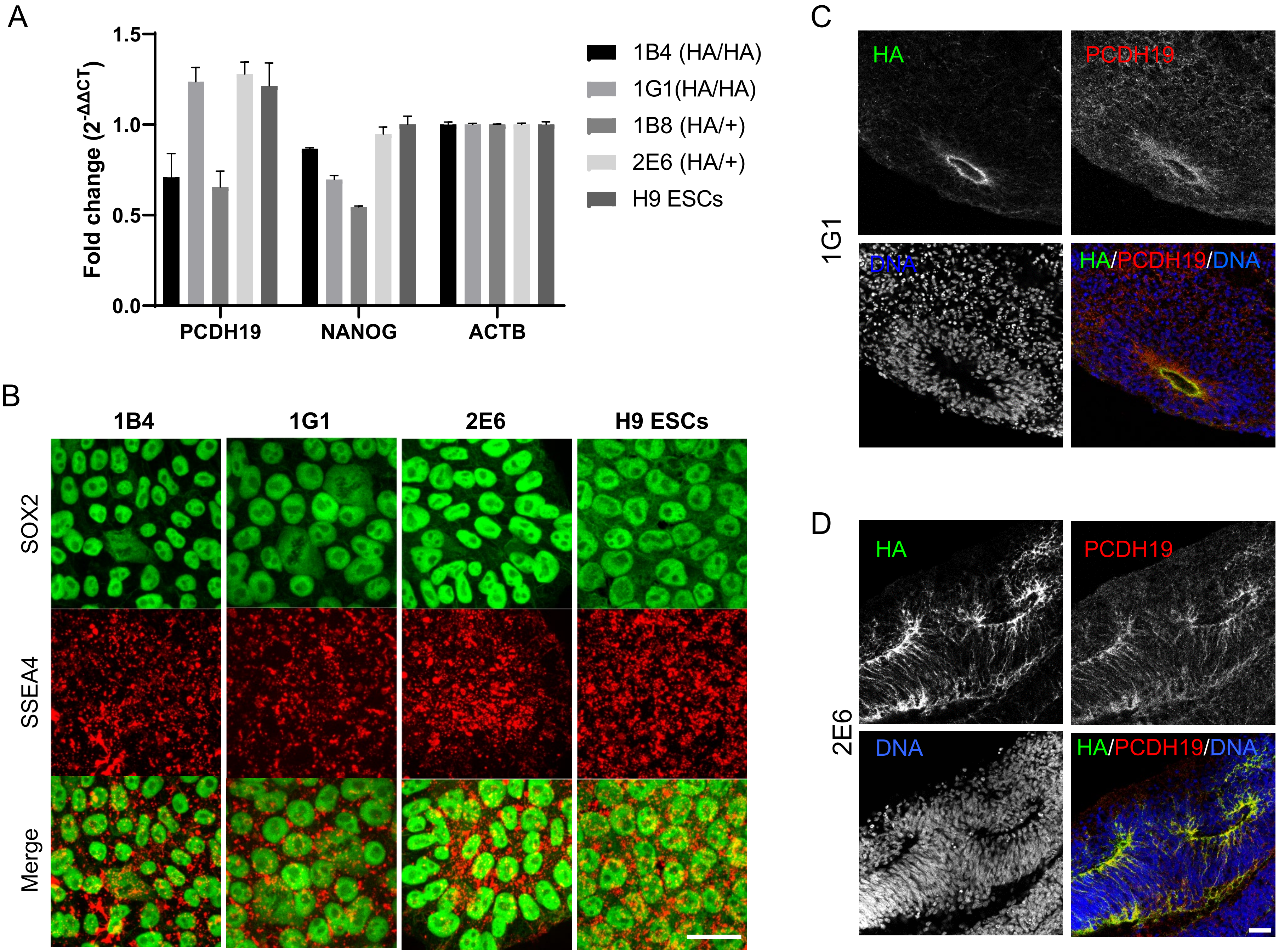

Supplementary Figure 2

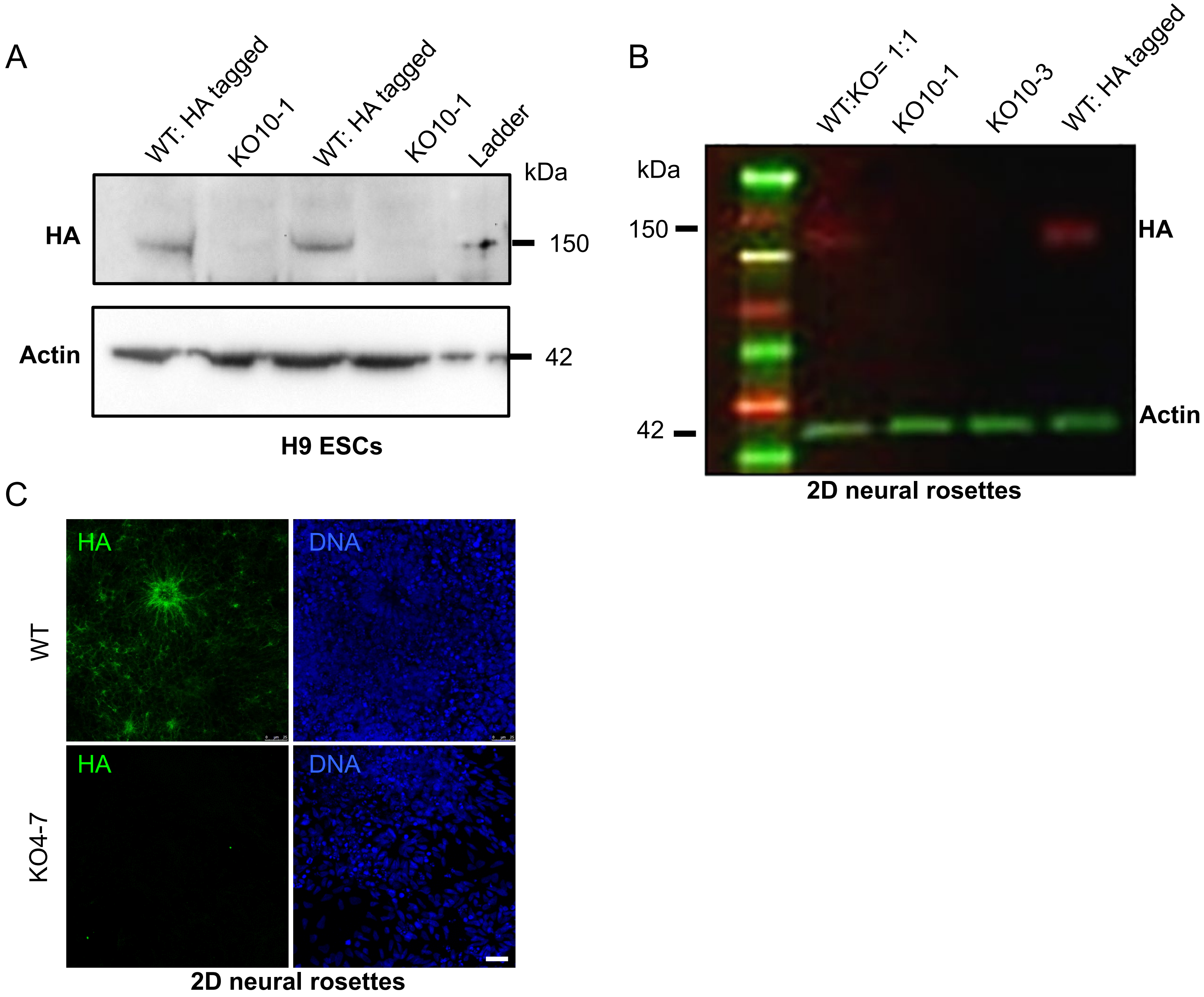

Supplementary Figure 3

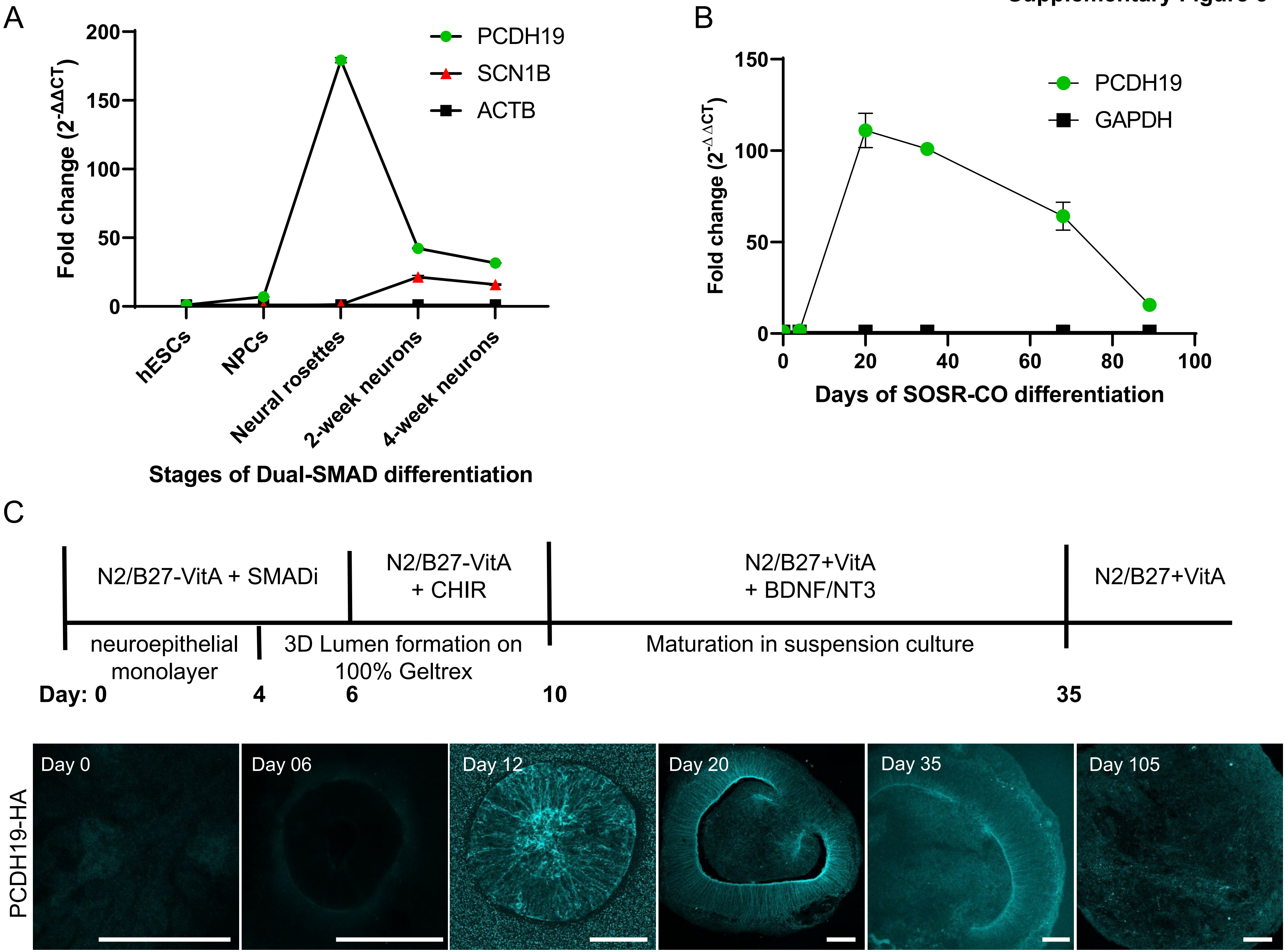

Supplementary Figure 4

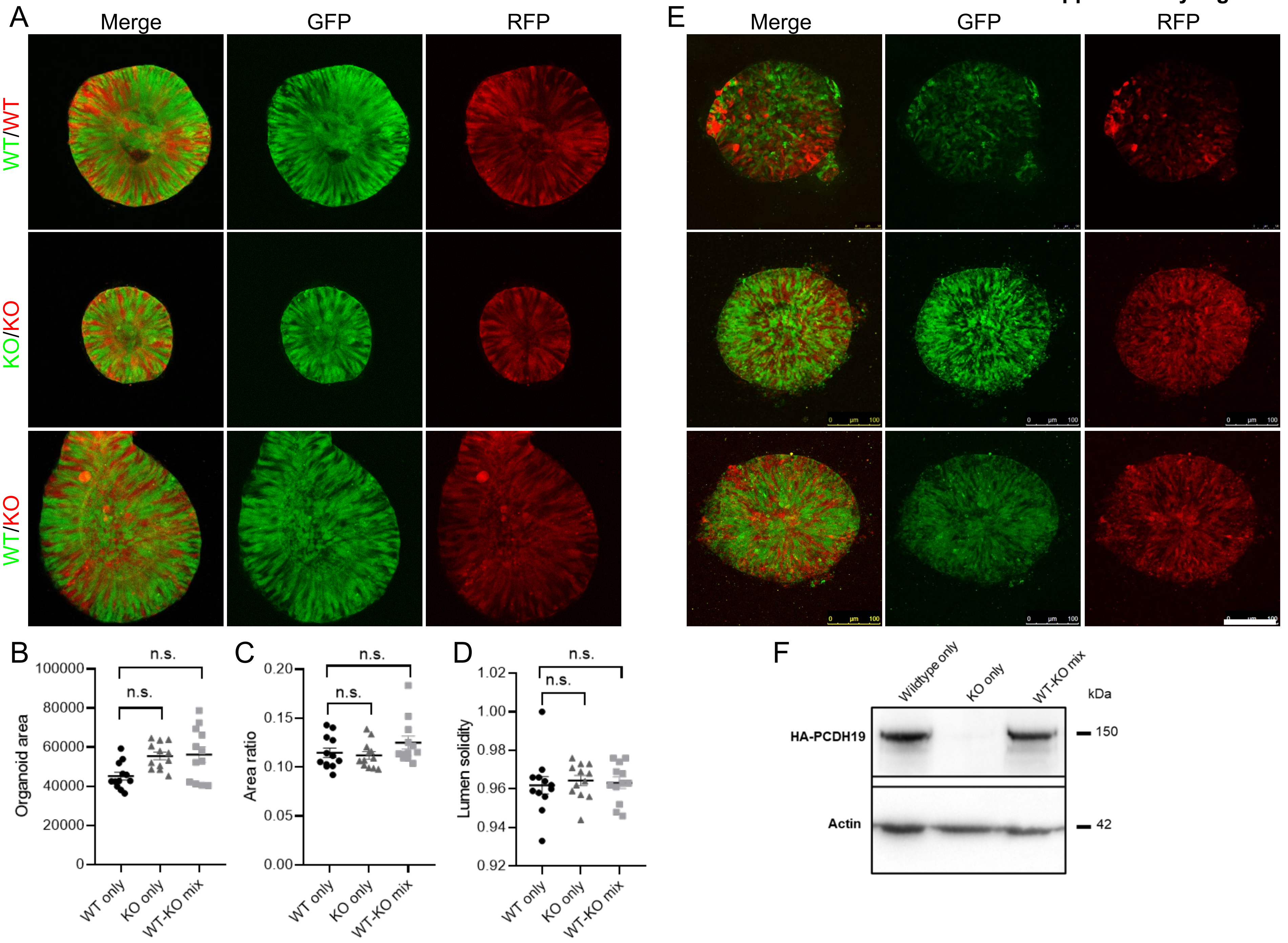

Supplementary Figure 5

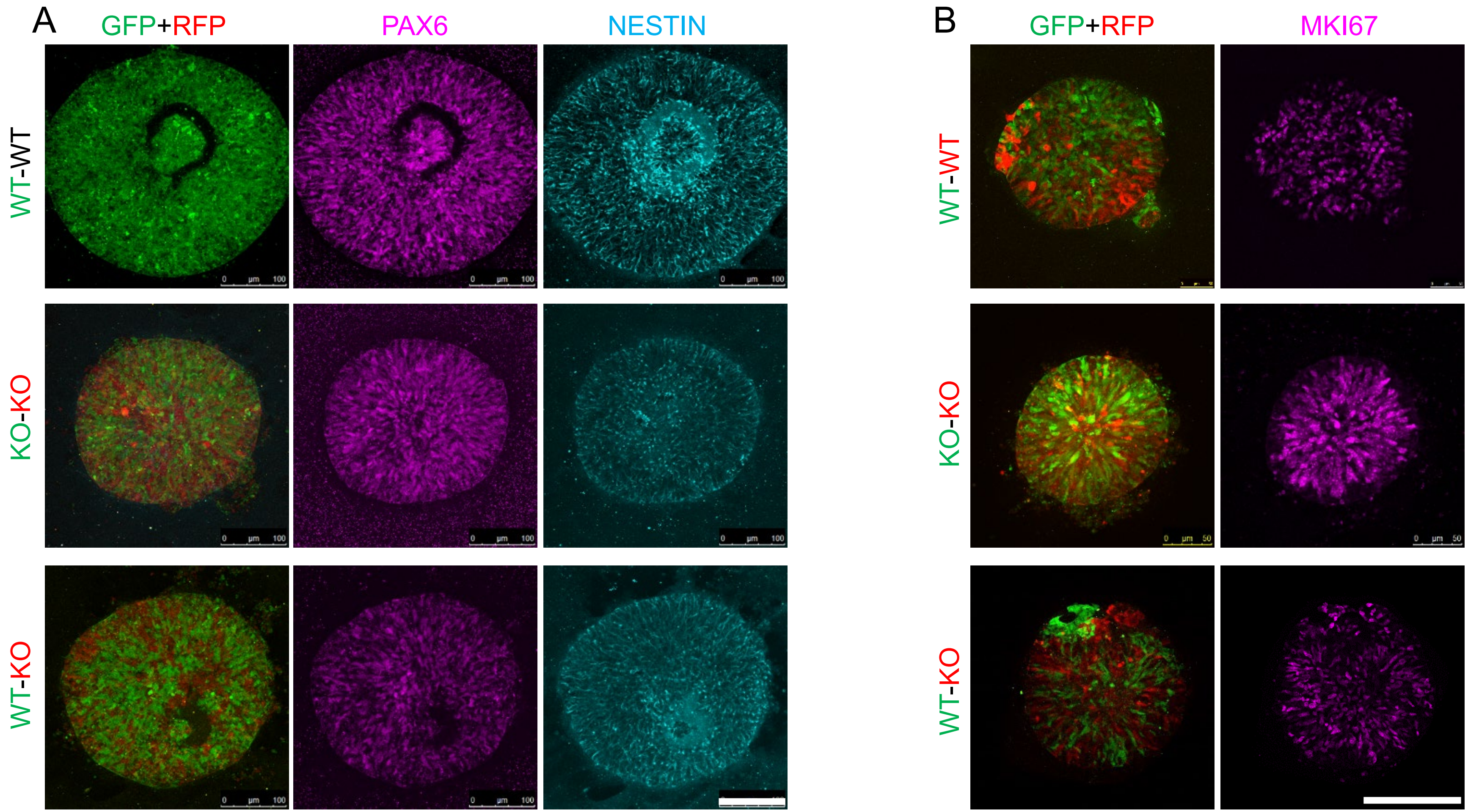

Supplementary Figure 6

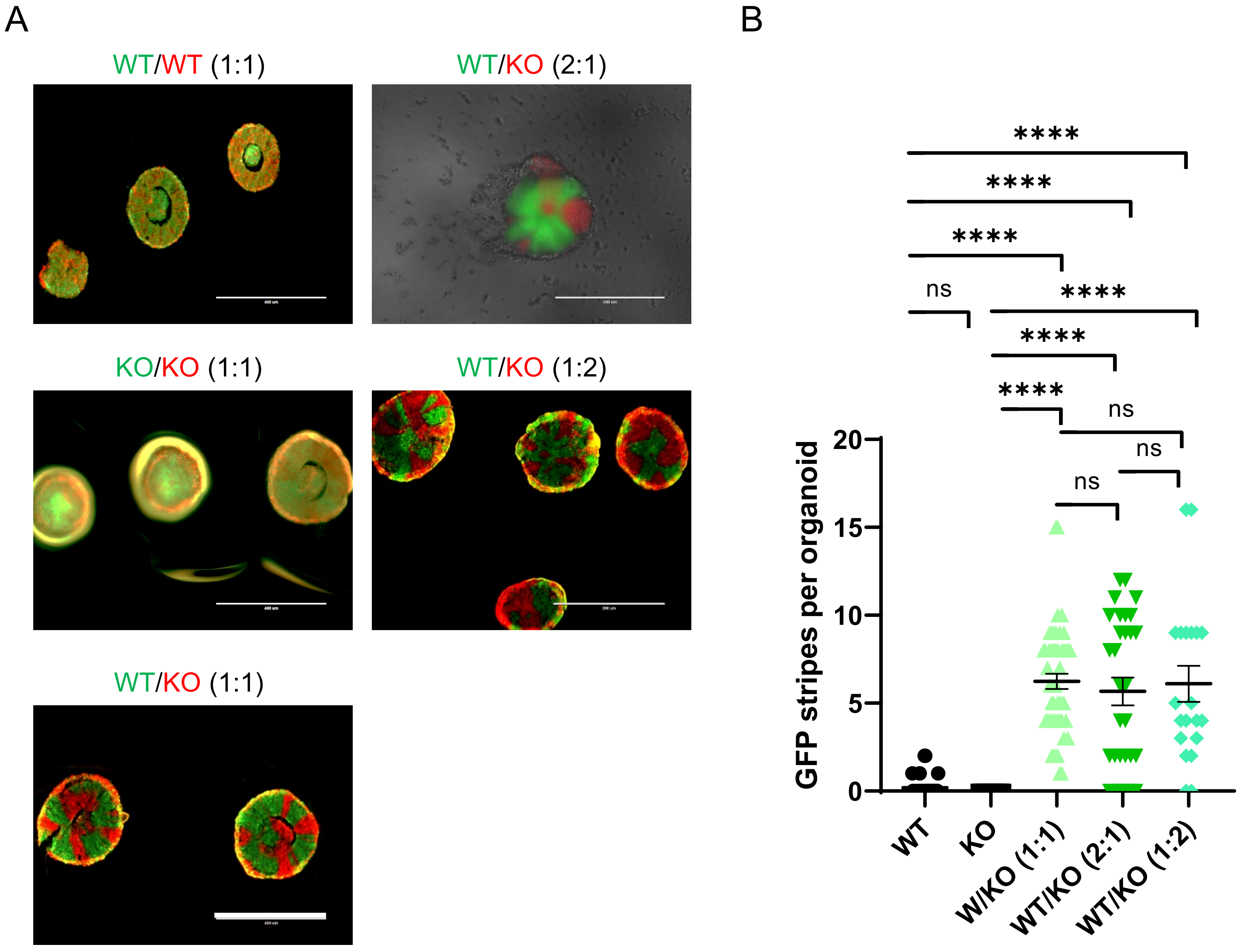

Supplementary Figure 7

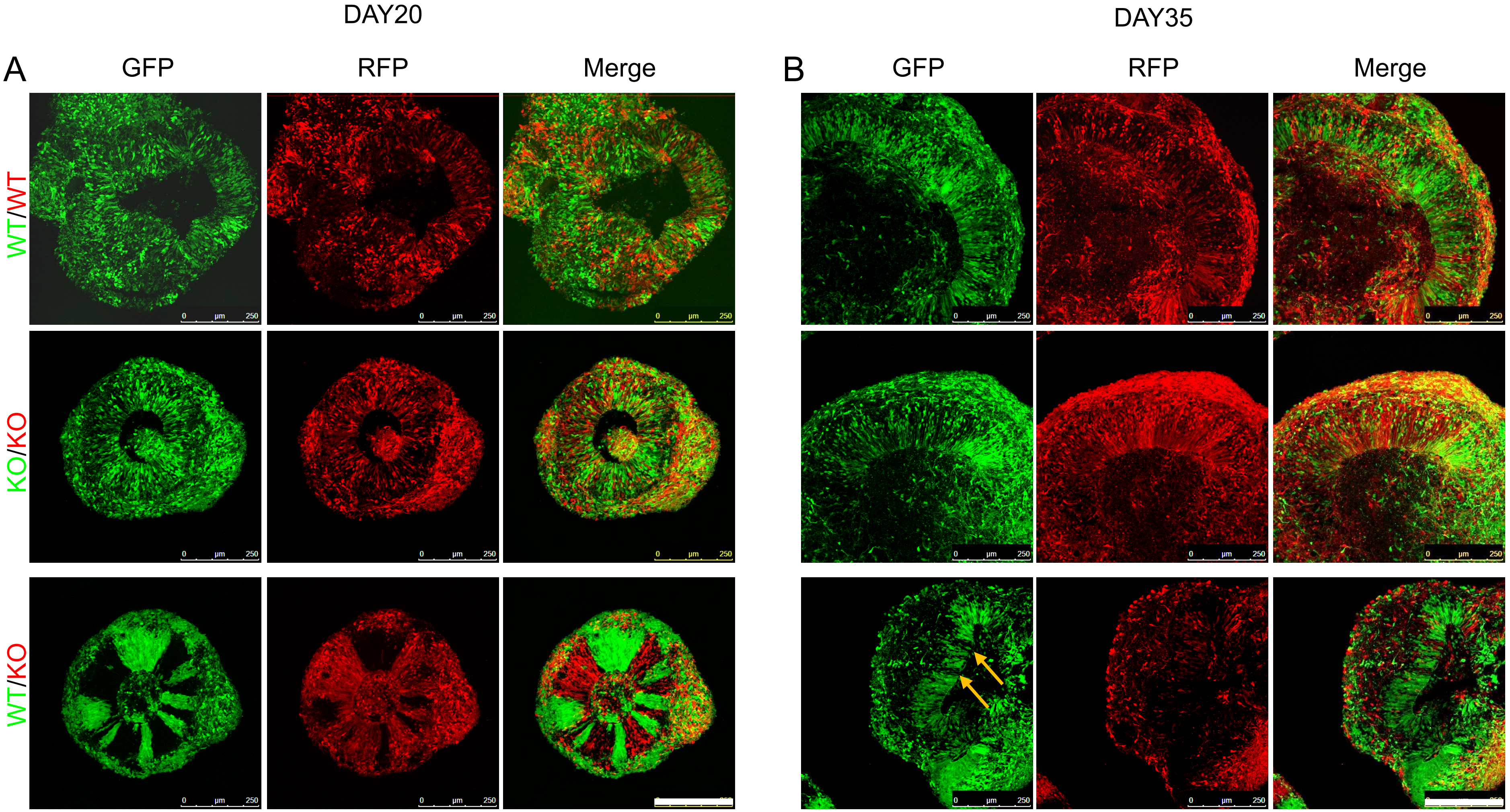

Supplementary Figure 8

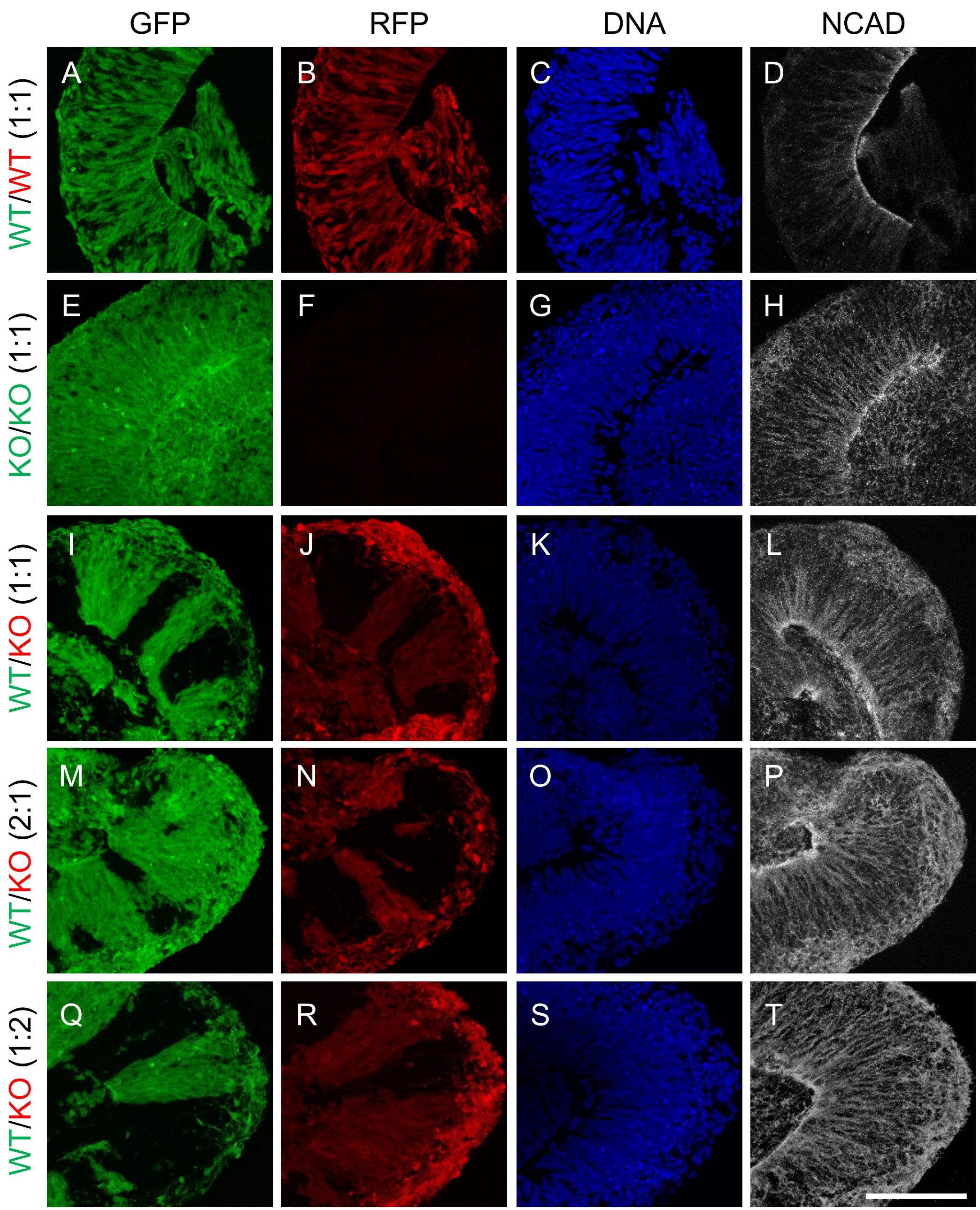

Supplementary Figure 9

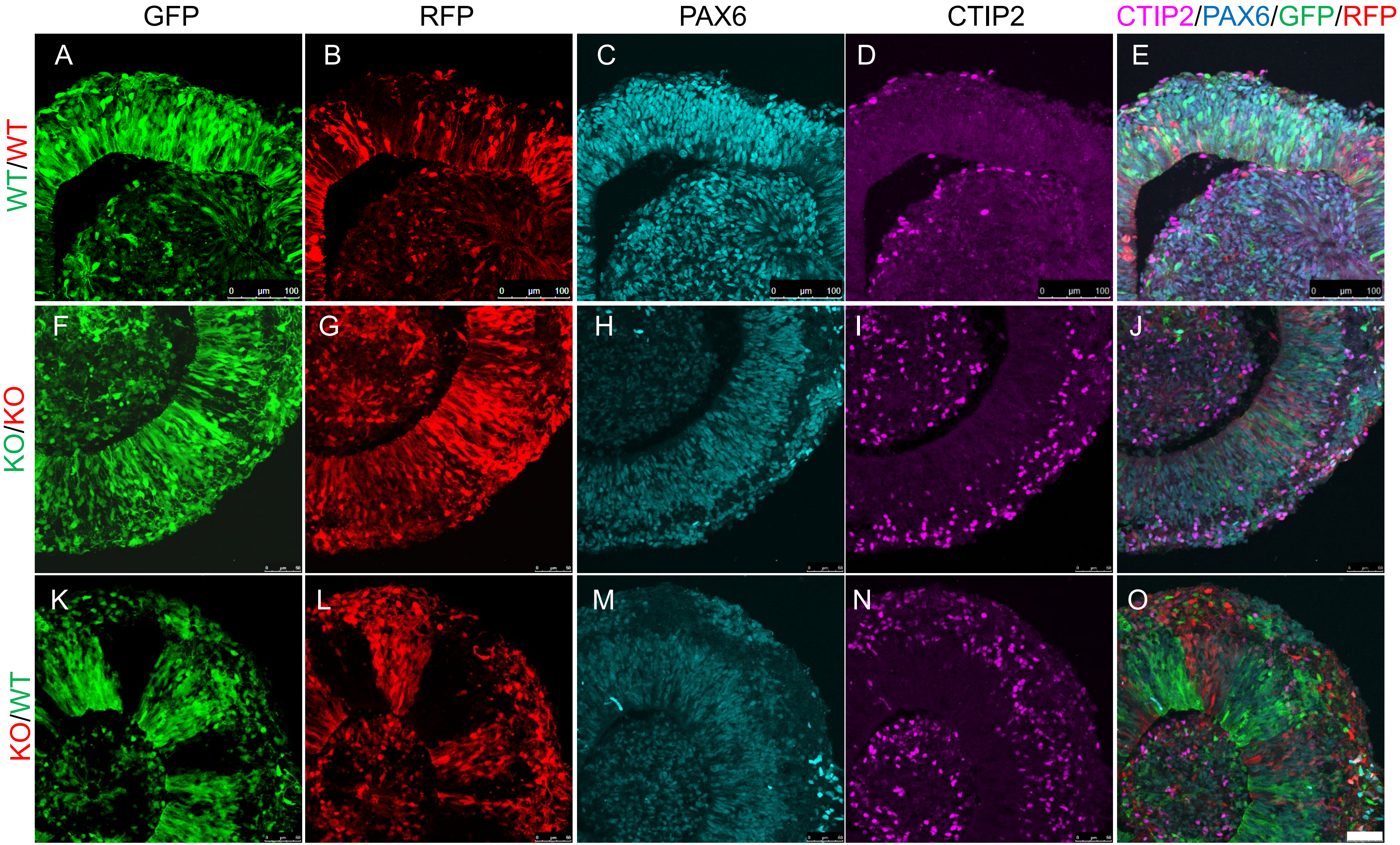

Supplementary Figure 10

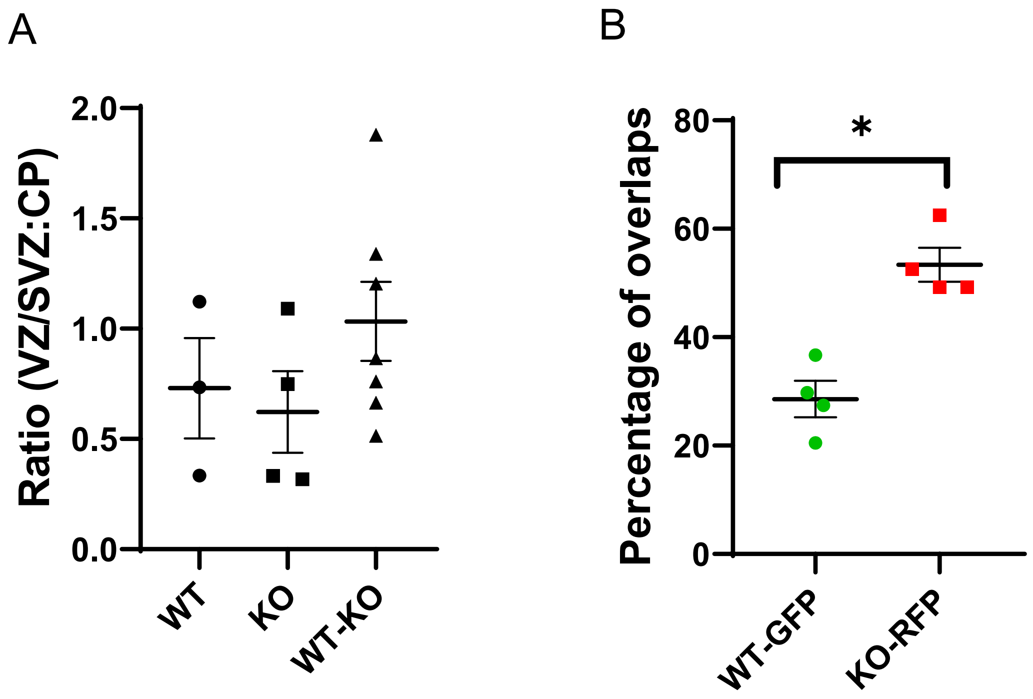

Supplement: Supplementary file 5 [file Data_Sheet_1.PDF]
